# A New Way to Look at the Data: Similarities Between Groups of People Are Large and Important

**DOI:** 10.1037/pspi0000154

**Published:** 2018-12-31

**Authors:** Paul H. P. Hanel, Gregory R. Maio, Antony S. R. Manstead

**Affiliations:** 1 School of Psychology, Cardiff University, and Department of Psychology, University of Bath; 2 School of Psychology, Cardiff University

**Keywords:** similarities, differences, gender, cross-cultural, education

## Abstract

Most published research focuses on describing differences, while neglecting similarities that are arguably at least as interesting and important. In Study 1, we modified and extended prior procedures for describing similarities and demonstrate the importance of this exercise by examining similarities between groups on 22 social variables (e.g., moral attitudes, human values, and trust) within 6 commonly used social categories: gender, age, education, income, nation of residence, and religious denomination (*N* = 86,272). On average, the amount of similarity between 2 groups (e.g., high vs. low educated or different countries) was greater than 90%. Even large effect sizes revealed more similarities than differences between groups. Studies 2–5 demonstrated the importance of presenting information about similarity in research reports. Compared with the typical presentation of differences (e.g., barplots with confidence intervals), similarity information led to more accurate lay perceptions and to more positive attitudes toward an outgroup. Barplots with a restricted *y*-axis led to a gross underestimation of similarities (i.e., a gross overestimation of the differences), and information about similarities was rated as more comprehensible. Overall, the presentation of similarity information achieves more balanced scientific communication and may help address the file drawer problem.

We are far more united and have far more in common with each other than things that divide us.

—Jo Cox (murdered British Member of Parliament; *Jo Cox maiden speech in the House of Commons*. (2016, June 17)).

Since Donald Trump won the election for the United States presidency in November 2016, an increase in racist attacks by 91% was reported in the first half of 2017 (*CAIR: Hate crimes against Muslims spike after Trump win*. (2017, July 18)). This increased racism parallels a sharp rise in the level of openly racist incidents after a narrow majority of British voters voted to leave the European Union in June 2016 (Furness, 2018; Versi, 2016) and the recent upsurge of support for right-wing parties across Europe (Wodak, KhosraviNik, & Mral, 2013). A key characteristic of racism is its focus on the idea that out-groups are different from and inferior to the in-group. Here, we suggest that quantitative social science may be inadvertently helping to foster these beliefs by focusing on differences between groups and neglecting to highlight stronger and important similarities.

For instance, if social scientists were comparing two groups of people with respect to moral attitudes, the researchers could describe either the differences or the similarities between the groups—or, indeed, both. Historically, the focus of social science research in general and psychological research in particular has been on the description of differences between groups. Over 90% of the published research findings in psychology describe statistically significant differences (Fanelli, 2010; Open Science Collaboration, 2015). Most of the inferential statistics and effect sizes used are only appropriate for measuring mean differences, and studies that “fail” to find significant differences are consigned to a literal or metaphorical file-drawer.

The lack of recognition that similarities matter is important because differences between groups with so-called “large” effect sizes can occur even when two groups are much more similar than different, as described below. Moreover, this possibility is applicable whenever people are clustered into groups based on a specific variable, including many demographic variables studied in psychology (e.g., gender, age, education, and culture). We illustrate these points in this article, beginning with a discussion of current approaches to comparing groups, before we describe the need to capture similarities between groups in a way that goes clearly beyond failing to reject the null hypothesis and statistical equivalence. Finally, we argue that the inclusion of information on similarities instead of an exclusive focus on differences has a range of important implications, such as more accurate perceptions of findings and more positive attitudes toward an outgroup.

## A General Test of Similarities and Methodology

**Figure 1 fig1:**
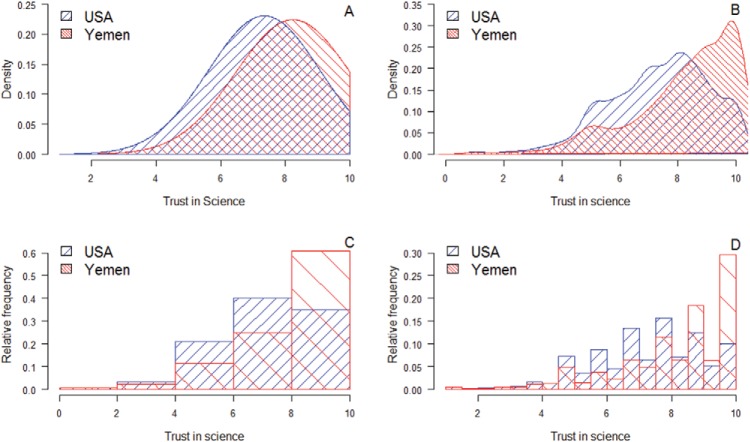
Different ways of presenting extent of Trust in Science in United States and Yemen, with *d* = .51 and 80% overlap of the distributions.

The presentation of research findings typically focuses on means, line graphs, bar graphs, *p* levels, or Bayes factors. These modes of presentation mainly focus on differences. When two or more groups are compared, researchers also tend to assume that a null difference indicates high similarity, whereas a statistically significant result reflects low similarity. However, while the null difference potentially affirms (or at least fails to refute) high similarity, a significant and/or large difference is *not* diagnostic of low similarity. Consider national differences in an important contemporary topic: trust in science. Figure 1 displays Americans’ (*n* = 2,202) and Yemenis’ (*n* = 911) trust in science, as reported in the World Value Survey (WVS; see below and online supplemental materials). Larger scale values (i.e., toward 10) reflect greater trust in science. As shown in Figure 1A, superimposing one density distribution on the other reveals a large overlap between the two samples. Most Americans chose response options that were also frequently chosen by Yemenis. Other ways of depicting the data also reveal large overlap. Figure 1B displays two Kernel distributions, which have been smoothed to the data. Figure 1C displays two histograms with five bars, and Figure 1D displays two histograms with 18 bars. All figures show high overlap, although a Welch’s *t* test of statistical difference reveals that Yemenis have significantly *greater* trust in science, *t*(1660.20) = 12.86, *p* < .0001, with a moderate effect size, Cohen’s *d* = .51, and the Bayes factor is >10^34^, using Rouder’s default JZS of *r* = .71 (cf. Rouder, Speckman, Sun, Morey, & Iverson, 2009). Overall, notwithstanding the moderate effect size, the vanishingly small *p* value, and the enormous Bayes factor, 80% of the responses are shared between the groups. The similarities are visibly far larger than the differences.[Fig-anchor fig1]

To make similarities more apparent in research reports, it is important to apply a general and easy-to-use method for quantifying similarity across diverse research domains. Although researchers have called for greater examination and discussion of similarities, especially in cross-cultural research (Berry, Poortinga, Breugelmans, Chasiotis, & Sam, 2011; Brouwers, Hemert, Breugelmans, & van de Vijver, 2004), a coherent approach to describing similarity has been absent. Our proposal is to avoid the problems associated with overreliance on both null hypothesis significance testing (NHST) and Bayesian statistics by supplementing them with information about similarities. To support this aim, we have reexamined a generally neglected measure of similarity and developed two new methods for assessing similarity.

**Figure 2 fig2:**
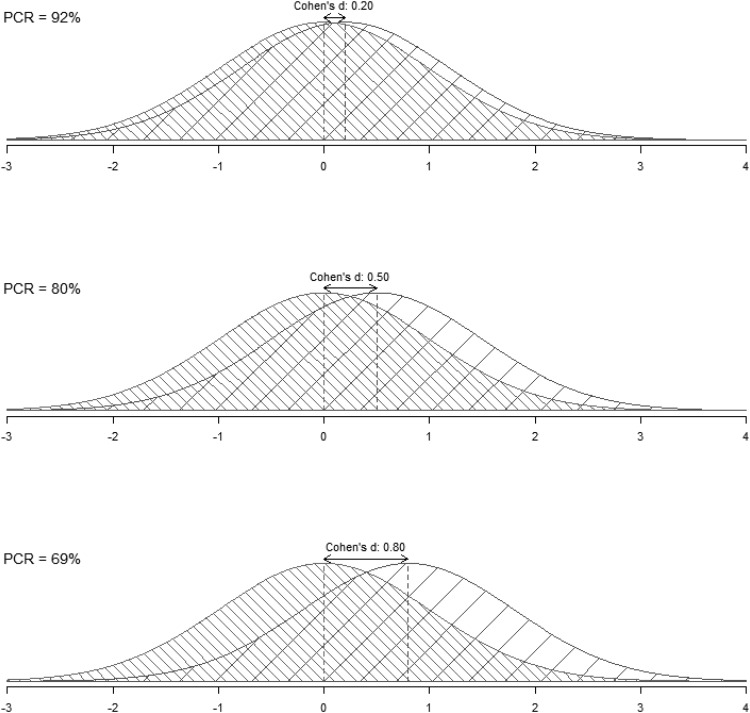
Illustration of the percentage of common responses (PCR) for different Cohen’s *d*s. The design of the figure was inspired by Kristoffer Magnusson’s interactive visualization on http://rpsychologist.com/d3/cohend/.

As background to understanding these new methods, it is important to consider current approaches to testing differences and to effect sizes representing differences. For example, a two-sample *t* test, one of the most commonly used tests in psychology, examines only the probability that two means are from the same population, while relying on the hypothesized distribution of the means and neglecting the actual distribution of the data. In other words, a *t* test can only help to ascertain whether the two means are likely to come from the same distribution; it does not allow any inferences about overlap across the breadth of the actual distributions. Similarly, one of the most popular indices of effect size, Cohen’s *d*, measures the differences between two means in units of variability (J. Cohen, 1988). A possible and often neglected application of *d* is that it can be transformed to yield an overlap coefficient (OVL), which allows one to draw conclusions about the distribution of all the data, not just the means, by estimating the percentage of overlap between two normal distributions (Inman & Bradley, 1989). In other words, the OVL helps one to assess similarity. Surprisingly, the OVL is rarely known and even missing in reviews of effect sizes for between-subjects designs (e.g., Lakens, 2013; Peng & Chen, 2014). For example, even a large effect size of Cohen’s *d* = .80 (J. Cohen, 1992) represents an overlap of 69%; a medium effect size of *d* = .50 represents an overlap of 80%; and a small effect size of *d* = .20 represents an overlap of 92% (see Figure 2).[Fig-anchor fig2]

In the online supplemental materials, we compare the OVL with six additional measures of similarities: the intraclass correlation (ICC[1]), the probability of superiority, a nonparametric version of the OVL, a new measure we developed to explicitly calculate the percentage of common scores (PCS), equivalence tests, and response surface analysis. We have developed the PCS to probe the robustness of the OVL, which relies on the assumption of normality. The PCS, which is described in more details in the online supplemental materials, can be interpreted as the percentage of one group that has the same scale responses as the other group, while controlling for different sample sizes and standardizing across response-scale lengths. Because the OVL and PCS are almost perfectly correlated (*r*s ≥ .96), we argue that it is useful to multiply the OVL by 100 and label this new index as the “percentage of common responses” (PCR), on the grounds that this simple percentage is a more concrete and familiar way to express similarity than an abstract decimal proportion representing “overlap.”

**Figure 3 fig3:**
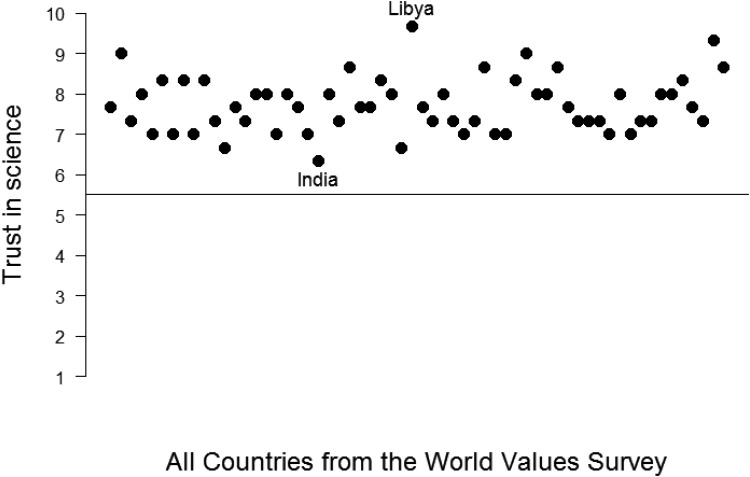
Median score of trust in science per country. The horizontal line represents the scale midpoint.

However, the PCR, like the conceptually related measures such as the percentage of common scores, the ICC[1], and the probability of superiority measure, neglects one other vital consideration for quantifying similarity: the *absolute* difference between the two means or medians, given a specific measurement scale. For example, small variances result in larger *d*s: If the *SD* is reduced by x, *d* will be increased by x. In other words, even small mean differences can result in large effect sizes and low estimates of similarity in distributions. For this reason, interpretation of the PCR needs to be supplemented with a measure of the absolute difference between two populations. To illustrate, consider once again the example of trust in science. To anticipate analyses reported below, we compared all 60 countries from the WVS with each other pairwise, resulting in 1,770 comparisons. On the basis of the “classical” approach that focuses on mean differences, one might conclude that countries differ considerably in their trust in science. The average *d* is 0.31, and the largest *d* is 1.60 (between India and Libya, *M* = 6.05, *SD* = 2.10, median = 6.33, vs. *M* = 8.92, *SD* = 1.53, median = 9.67). Many of the mean differences between any two given countries are statistically significant, which is not surprising given an average sample size of 1,438 per country. A few of the mean differences also seem appreciable when considered alongside the percentage of common responses for some countries. For example, the PCR between India and Libya is only 42. However, the picture looks different when the median scores are considered. Participants in these two countries (and indeed in each of the 60 countries) scored significantly higher on average than the midpoint of the 10-point Likert scale (see Figure 3). In short, people in *all* of the nations exhibit a high trust in science.[Fig-anchor fig3]

This similarity in central tendency can be captured by calculating the absolute effect (AE), which we define simply as the median difference expressed as the percentage of the largest possible difference: 100 × (median Group A—median Group B)/(scale maximum—scale minimum). If the median difference is 0.50, measured on a scale ranging from 1 to 6, the AE would be 100 × .5/(6–1) = 10. This statistic is a generalization of the percentage of the maximum possible score metric (POMP; P. Cohen, Cohen, Aiken, & West, 1999), which has been designed to standardize single case observations across scales (e.g., school grades from different countries) to a two-group comparison. Thus, the AE is the difference between two POMP scores with the medians used as observations. In contrast to distribution-dependent measures such as Cohen’s *d* or PCR, the AE is independent from the type of distribution and the variance while taking the scale range into account. While Cohen’s *d* is the mean difference relative to the *SD*, the absolute effect is the median difference relative to the scale range. In the current example, calculating the AE reveals that the proportion of the median difference in trust in science between countries is rather small (median AE = 7; the maximum AE of 37 is between India and Libya, 95% confidence interval, CI [37, 41]).^1^

Despite their conceptual independence, PCR and AE are negatively related in the data we examined (see below). In combination, these indices allow researchers to describe whether two groups are more similar or different: If the PCR is >50 *and* AE <50, similarities between two groups are larger than the differences. If the PCR is <50 and the AE >50, the two groups are more different than similar. We argue that it is important to take both effect size estimates into account to determine whether groups are more similar or more different because a large PCR can arise when the *SD*s are rather small while the means and medians are still on one side of the scale, as in the example displayed in Figure 3 for the variable trust in science. Thus, there is a clearer case for relatively strong group differences when the distributions show less than 50% overlap and on average the groups fall on a different side of the scale. Of course, it is possible that the PCR is less than <50 and the AE is less than 50. In this case, we would argue that it is undetermined whether groups are more different than similar. This situation is akin to the stalemate that occurs for other statistical procedures that rely on more than one statistic. For example, in structural equation modeling, it can happen that one set of fit indices indicates reasonable fit (e.g., comparative fit index [CFI]/Tucker-Lewis Index [TLI] ≥ .90), while the other set of indices suggests a bad fit (e.g., Root Mean Square Error of Approximation ≥ .10; root mean square error of approximation). Nonetheless, as noted above, the empirical relations between PCR and AE make this situation comparatively unlikely.

## Implications of Documenting Similarities

We argue that a stronger focus on similarities between groups has a range of important implications, such as improved intergroup attitudes, more accurate perceptions of effect sizes, more balanced scientific communication, and reduction of the metaphorical file drawer problem. We discuss the first two implications in this subsection, because we will directly test them in the present article, whereas the other implications are outlined in the General Discussion.

Similarities between groups are often ignored in the reporting of results, even though they have a fundamental relevance to the “take-home” message for readers. For example, an empirical report could highlight the fact that Americans trust science less than Yemenis, but this emphasis would miss the fact that both groups trust science to a great extent. This omission is potentially significant: Research in social cognition has found that focusing on similarities versus differences fundamentally affects how people interpret information about others (Mussweiler, 2003; Suls & Wheeler, 2000). Further, abundant research has found that highlighting similarities between groups improves interpersonal and intergroup attitudes (Brown & Abrams, 1986; Byrne, 1961; Montoya, Horton, & Kirchner, 2008; Pilkington & Lydon, 1997). Neglect of similarities in scientific reporting directs readers’ reasoning in a way that is likely to exacerbate the imputation of differences.

We further argue that highlighting similarity information will result in a more accurate perception of effect sizes. This is because similarity information facilitates inferences about the whole distribution of responses rather than some truncated information (e.g., means and *SE*s). Also, the PCR should be easier to interpret because it relies on one of the most often used statistics in everyday life, *percentage*. In contrast, the interpretation of Cohen’s *d* as “standardized mean difference” is more technical and abstract in relation to the data. (Study 2 demonstrates the implications of this difference for ease of comprehensibility.)

Similarity information may be more informative particularly when results are visually presented. For example, it was argued that a barplot with a restricted (truncated) *y*-axis causes an effect to appear larger than it actually is (Huff, 1954), whereas superimposed normal distributions or histograms (see Figure 1) provide a more complete picture of the responses. The latter visual methods provide information about the full distribution of responses and not only sample-size dependent information of the distribution (e.g., *SE*, CI). Indeed, it was recently argued that graphs in biology rely too often on summary statistics, as in bar and line graphs (Weissgerber, Milic, Winham, & Garovic, 2015). Instead, the authors recommended the use of graphs containing distributional information such as unidimensional scatterplots. This call was also made by Lane and Sándor (2009) who suggested boxplots, back-to-back histograms, or back-to-back stem-and-leaf displays. In the current research, we tested directly whether graphs that highlight similarities are considered to be more informative and allow readers to form more accurate estimations of the effect sizes than barplots with restricted *y*-axes.

We focus here on comparisons of barplots with restricted *y*-axes because these are still the dominant method of data presentation in psychology, despite Huff’s observation over 60 years ago (Huff, 1954). The majority of graphs in two leading psychological journals were bar graphs “and only about 10% of the graphs showed distributional information beyond central tendency” (Sándor & Lane, 2007; as cited in Lane & Sándor, 2009, p. 240). This is not surprising given that a restricted *y*-axis highlights what most researchers are testing for, mean differences, more clearly than a graph with an unrestricted *y*-axis. Thus, it is important to test for potential detrimental effects of restricted *y*-axis graphs in comparison with other formats that provide information about distributional overlap.

## The Present Research

In Study 1, we provide a broad demonstration of the utility and importance of calculating similarity indices by testing for similarities across six categories: gender, age, education, income, countries, and religious denomination. We tested for similarities on 22 dependent variables, including human values, moral attitudes, and trust in other people. These choices were made with an eye to examining variables that have often been reported as showing important differences between people. If there is evidence of high similarity in these tests, the data would provide an important caveat to prior conclusions about differences.

In large, representative international samples, the present research addressed this issue using the new indices, PCR and AE, together with Cohen’s *d* and the percentage of common scores (PCS, see below and online supplemental materials) for the purpose of comparison. Within a given category (e.g., countries), we compared each group with each other (e.g., Americans with Yemenis) to compute all the statistics. For example, we compared 60 countries pairwise, resulting in 1,770 comparisons for each statistic and variable. For the remaining five categories, we conducted 148 pairwise comparisons: 21 for religious denominations, 1 for gender (male vs. female), 36 for education, 45 for income, and 45 for age. In total, we conducted 168,784 pairwise comparisons. CIs for the PCRs and AEs were bootstrapped.

We expected to find large correlations between the PCR, AE, and the other measure of similarity, PCS. We then explored potential differences in levels of similarity across our comparisons. For example, we tested whether people were less similar to each other when clustered in different income groups than when clustered in countries, as Greenfield (2014) proposed. Furthermore, we explored which variables show more similarities and which variables show fewer similarities across all six categories.

Studies 2–5 were designed to test several implications of emphasizing similarities rather than differences when reporting scientific findings. In Study 2, we tested whether highlighting similarities when presenting comparisons between groups leads to a more accurate estimate of the actual degree of similarity between groups than when differences are highlighted. Further, we were interested in whether certain types of graphs are rated as more comprehensible than others, and whether the PCR and PCS are easier for a layperson to understand than Cohen’s *d*. Studies 3 to 5 replicated and extended Study 2 by testing whether emphasizing similarities also leads to more positive attitudes toward members of an outgroup. For this, we relied on archival data (Studies 3 and 4) and on influential studies within psychology.

The R code used to obtain the results of Study 1 and most of the results of Studies 2–5 along with the data of Studies 2–5 can be found at https://osf.io/bxu5m/ (the dataset used in Study 1 is publicly available at http://www.worldvaluessurvey.org).

## Study 1

### Method

#### Participants

We used the most recent version of the WVS at the time of conducting our analyses (6th round; April 2015), which includes 86,272 participants (51.20% female) from 60 countries with a mean age of 41.68 years (*SD* = 16.58).

#### Material

Our independent variables were gender, age, education, income, country of residence, and religious denomination. Gender was measured dichotomously as male or female. The continuous variable year of birth was divided into 10 equal sized groups, ranging from “born in 1946 or before” to “born in 1991 or later” to allow pairwise comparisons. Education was measured on a 9-point scale ranging from 1 (*no formal education*; *n* = 4,604) to 9 (*university-level education with degree*; *n* = 14,260). To estimate income, participants were asked to indicate their household net income, relative to the country-specific income distribution, on a 10-point scale from 1 (*lowest group*) to 10 (*highest group*). For country, we used all 60 countries in the WVS. More than 1,000 participants indicated that they belong to one of the following seven religious denominations: Buddhism (*n* = 3861), Evangelical (*n* = 1411), Hindu (*n* = 1742), Muslim (*n* = 21,230), Orthodox (*n* = 8505), Protestant (*n* = 5562), and Roman Catholic (*n* = 14,921).

The 22 dependent variables included the 10 value types postulated by Schwartz (Schwartz, 1992). They were measured in the WVS with a short version of the Portrait Value Questionnaire (Schwartz, 2003; Schwartz et al., 2001), containing one item per value type. This measure asks participants to rate the extent to which each description (portrait) of another person is similar to them. Example descriptions include “Adventure and taking risks are important to this person; to have an exciting life” (stimulation) and “It is important to this person to be rich; to have a lot of money and expensive things” (power). Responses were given on a 6-point Likert scale ranging from 1 (*very much like me*) to 6 (*not like me at all*). We chose the other 12 variables by considering the scales within the WVS, the findings of previous studies, and the results of principal component analyses (PCAs). For instance, although trust in other people had been measured in previous studies with a single scale (Jen, Sund, Johnston, & Jones, 2010), we created two factors from the six items, based on the results of a PCA (varimax rotation). All six items were answered on a 4-point scale from 1 (*trust completely*) to 4 (*do not trust at all*). The first factor represents trust in strangers, and is based on trust in people met for the first time, trust in people of a different religion, and trust in people from a different country (α = .79). The second factor represents trust in people with whom one is close (family, neighbors, and people known personally; α = .58). The correlation between the two factors was of medium size, *r*(83,962) = .35. Other variables were understanding of democracy (6 items, α = .74), trust in political institutions (6 items, α = .87), perceived respect for the elderly in society (3 items, α = .71), ageism (4 items, α = .60), trust in science (3 items, α = .73), skepticism toward science (3 items, α = .56). Trust in science was uncorrelated with skepticism toward science, *r*(83,686) = −.07. Further, the items of the Morally Debatable Scale (Harding & Phillips, 1986) were divided into three subscales after a PCA (cf. Vauclair & Fischer, 2011): attitudes toward personal-sexual behaviors (e.g., justifiability of homosexuality, 7 items, α = .89), dishonest-illegal behaviors (e.g., stealing, 5 items, α = .83), and domestic violence (e.g., a man beating his wife, 3 items, α = .78). Higher scores on these attitude scales signify greater agreement. Finally, political attitude was measured with a single item, on which participants could indicate their position on a left-right political dimension from 1 (left; *n* = 3459) to 10 (right; *n* = 5354), with most participants identifying themselves as in the middle (5 items, *n* = 19,005).

### Results

We summarize important findings for each category, followed by comparisons between all categories. Detailed tables of results can be found in the online supplemental materials. The analyses focus on the two similarity measures we propose, PCR and AE. As expected, these were negatively correlated, *r*(130) = −.65, *p* < .001. Of importance, the PCR, despite being a parametric measure, is robust against violations of normality assumptions: There is a large correlation between PCR and PCS (*r*s = .95 to .98), a nonparametric index of similarity described in the online supplemental materials, and the average difference between them is only 2%.^2^

#### Findings within each category

**Figure 4 fig4:**
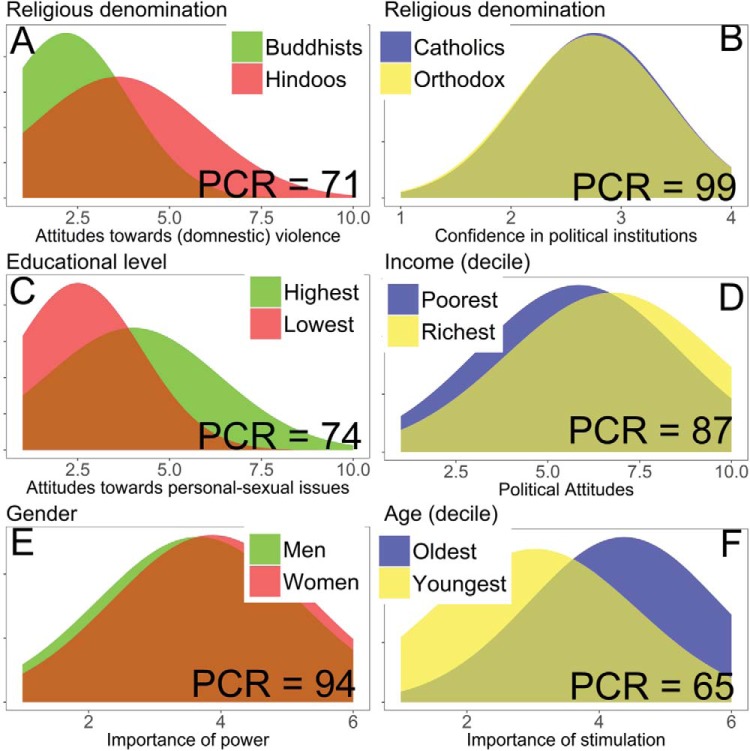
Selected pairwise comparisons for the six categories.

As expected, there were high levels of similarity within categories. For all variables, the median PCR between two groups is 95.00 (*M* = 93.30, *SD* = 5.37, range = 71–100), and the AE is 2% (*M* = 5.45%, *SD* = .07, range = 0–20), indicating large similarities. Only 274 out of 41,821 pairwise comparisons (0.66%) for both effect sizes combined revealed a PCR of less than 50% and an AE of more than 50%. All of these 274 cases were found in the *country* category (see Supplementary Table 1). For illustration, Figure 4 shows selected pairwise comparisons for five of the six categories. These comparisons were partly selected based on their relevance to previous literature. For example, Schwartz and Rubel (2005) found that the largest mean difference between women and men for human values (Schwartz, 1992) can be found for power (Figure 4E), whereas Robinson (2013) found one of the strongest age effects for stimulation (Figure 4F). Comparisons for country can be found in Figures 1, 7, and 10.[Fig-anchor fig4]

##### Countries

Countries were less similar to each other than were the groups in the other categories. The average PCR across all 22 variables was 84 (range = 71–90) and the average AE was 14% (range = 0 to 20%). The two variables with the smallest PCRs and largest AEs were moral attitudes toward personal-sexual issues and moral attitudes toward (domestic) violence (PCRs = 6, 95% CI [4, 7], and 28, 95% CI [23, 33]; AEs = 67%, 95% CI [65, 69] and 41%, 95% CI [41, 41]): Participants from Pakistan considered liberal personal-sexual behaviors (e.g., abortion, homosexuality) least justifiable (*M* = 1.63, median = 1.14), whereas those from the Netherlands (*M* = 6.69, median = 7.14) and Sweden (median = 7.14) found them most justifiable. Participants in 28 countries reported that (domestic) violence is not justifiable (*M* < 2, medians = 1), whereas participants from Rwanda found it relatively more justifiable (*M* = 4.50, median = 4.67; the PCR reported above is from the comparison of Rwanda with Sweden: Sweden is one of the 28 countries with a median of 1 for attitudes toward domestic violence). However, the average Rwandan reported that (domestic) violence is less justifiable than justifiable, as did the average respondent from the other 59 countries (Supplementary Table 1); the largest AE for these 1,700 pairwise comparisons was 41%.

##### Religious denomination

Pairwise comparisons were made for seven religious denominations. The average amount of similarities across 22 dependent variables was large (PCR = 91, range = 84–96) and the average AE was 5% (range = 0 to 20%). The smallest PCR was with respect to moral attitudes toward (domestic) violence (PCR = 66, 95% CI [63, 68]; AE = 22%, 95% CI [19, 22]), with Evangelicals reporting related behaviors as less justifiable (*M* = 1.77, median = 1) than Hindoos (*M* = 3.59, median = 3).

##### Income

The amount of similarities was again large with an average PCR of 96 (range = 92–98) and an average AE of 4% (range = 0 to 20%). The smallest similarity occurred for political attitudes (PCR = 79, 95% CI [76, 82]; AE = 22%, 95% CI [22, 33]), with participants in the second lowest income group having a stronger proleft attitude (*M* = 5.44, median = 5) than those in the highest income group (*M* = 6.77, median = 7).

##### Education

Similarities between educational groups were large: The average PCR was 96 (range = 91–98) and the average AE was 2% (range = 0 to 10%). The smallest similarity was in moral attitudes toward liberal personal-sexual behaviors (PCR = 74, 95% CI [73, 75]; AE = 19%, 95% CI [17, 19]), with participants having the lowest educational level reporting them to be least justifiable (*M* = 2.50, median = 2.00), and those with the highest educational level reporting them to be relatively more justifiable (*M* = 3.94, median = 3.67).

##### Gender

The amount of similarity was again large with an average PCR of 97 (range = 90–100) and an average AE of 3% (range = 0 to 20%). The smallest similarity was for the value type *stimulation* (PCR = 90, 95% CI [90, 91]; AE = 20%, 95% CI [20, 20]), with women reporting it to be less important (*M* = 3.87, median = 4) than men did (*M* = 3.49, median = 3), with lower values indicating greater importance.

##### Age

The amount of similarity was again large with an average PCR of 96 (range = 88–99) and an average AE of 4% (range = 0 to 20%). The smallest similarity was for the value type *stimulation* (PCR = 65, 95% CI [64, 66]; AE = 40%, 95% CI [40, 40]), with those born in 1946 or earlier valuing stimulation less than those born after 1990 (*M* = 4.36 vs. 3.02, medians = 5 vs. 3, respectively, with lower values indicating greater importance). Although we cannot distinguish between age and cohort effects in these comparisons, the issue is tangential to our current focus.

#### Comparisons between all categories

To identify the categories for which similarities were the largest, we compared the PCRs and AEs between the six categories across the 22 variables. That is, we treated the variables as cases, and subjected the PCRs and AEs to 6-level (category type) repeated-measures analysis of variances (ANOVAs). Because of violations of the sphericity assumption, the Greenhouse-Geisser correction was applied. Both the PCRs (*F*(2.46, 51.72) = 85.57, *p* < .001, $\eta_{p}^{2}\;$ = .80) and the AEs (*F*(3.80, 79.81) = 15.63, *p* < .001, $\eta_{p}^{2}\;$ = .43) differed significantly between categories. For both statistics, pairwise comparisons revealed that countries were less similar to each other than were the groups in each of the other categories (all *p*s ≤ .001). Countries were less similar to each other (*M*_PCR_ = 84, *M*_AE_ = 14) than were religious denominations (*M*_PCR_ = 90, *M*_AE_ = 6), income groups (*M*_PCR_ = 96, *M*_AE_ = 4), educational groups (*M*_PCR_ = 96, *M*_AE_ = 2), women and men (*M*_PCR_ = 97, *M*_AE_ = 3), and age cohorts (*M*_PCR_ = 96, *M*_AE_ = 4).

A second category where the degree of similarity was somewhat lower compared with the remaining four categories was religious denomination (*p*s < .001), but this applied only for the PCR measure; the differences for AE were less consistent (see Supplementary Tables 1 to 6). The difference in PCR between religious denominations and the remaining four categories ranged between 5.41 and 6.86, indicating smaller levels of similarities between religious denominations, relative to those for income, education, gender, and age.

### Discussion

The objective of Study 1 was to report the magnitude of similarities between groups of people across six important psychological categories and 22 topical variables. Across all variables, the average PCR was 93 when we compared people from different genders, ages, educational attainments, incomes, countries, and religious denominations. This high level of similarity was corroborated by the AE. The median AE revealed that the difference between any two groups across all categories and variables was only one-twentieth of the possible difference on the scale in question. Furthermore, these findings were replicated in a similar study of a different dataset, containing responses from 29 European countries (*N* = 54,082), as reported in the online supplemental materials.

Countries were on average less similar to each other than were the groups in other categories. This challenges the claim that countries are more similar to each other than are people with different socioeconomic status (Greenfield, 2014). The lowest degree of similarity across all comparisons was for moral attitudes toward personal and sexual issues. This finding is broadly consistent with Graham et al.’s (2011) evidence that the largest differences between various groups of people—mainly conservatives and liberals—is on the moral dimension *purity*, which is closely related to moral attitudes toward personal and sexual issues.

Notwithstanding the evidence of smaller similarities between countries than between groups in the other categories, the high average degree of similarity between countries supports the universalism claim in cross-cultural research, which holds that values, attitudes, and beliefs are weakly influenced by cultural factors (Berry et al., 2011). At the same time, however, we can now be more confident that country has a relatively strong influence on variables like human values, independently of religious denomination, education and income level, or age distribution. If country differences were mainly because of differences in religious denomination, education level, or age distribution, we would have found groups in these categories to be less similar to each other than countries. By contrast, we found that countries were less similar to each other than were groups in these other categories.

It is important to keep in mind that we could have presented all of our comparisons without PCR and AE data and focused only on effect sizes quantifying differences (e.g., *d* scores). For example, we could merely have concluded that differences between countries are larger (*M*_d_ = .39) than differences between groups in the other categories (for the Cohen’s *d*s see online supplemental materials). Such a focus would tell a different story, focusing on large *d*s, and ignoring the large number of small effects. Not only would such an approach result in most of the results attracting little attention because of small effect sizes (i.e., ending up in a virtual file-drawer), but a focus on large effect sizes would also have obscured the fact that the similarities are very large in many cases. Adding PCR and AE to the analyses helps to put the interpretation of *d*-scores, *p* values, and/or Bayes factors into perspective and increases the interpretability of the findings, as Study 2 demonstrates.

We are not suggesting that differences are unimportant. Instead, we argue that adding information about the amount of similarity helps one to arrive at a more balanced and intuitive understanding of the effect size than would be the case if one is only provided with the difference test effects sizes. In Studies 2–5, we test this argument by presenting data visually in ways that emphasize either similarities or differences. We use also these studies as an exercise in demonstrating how similarities can be reported and interpreted alongside differences. These studies focus on similarity indices in the context of pairwise comparisons, because to the best of our knowledge no similarity index for more complex designs (e.g., two-way ANOVAs) has yet been developed.

## Study 2

Study 2 focused on whether reporting similarities compared with differences leads to more accurate perceptions of effects in lay reading of reports. Specifically, we explored which of three ways of displaying the same results, superimposed normal distributions, barplots with CIs, and superimposed histograms (see Figure 3), yields a more accurate estimation of similarities and is perceived as clearer and more informative. We selected superimposed normal distributions and histograms rather than boxplots, which were recommended as alternatives to bar- and line-plots (Lane & Sándor, 2009), and rather than violin-, ridgeline-, pirate-, or other types of plots, which display the full distribution of the data, because box-, ridgeline-, or violin-plots are usually presented next to each other and are not superimposed. Consequently, they do not highlight similarities to the same extent as the two types of graphs we are using. This does not mean that we consider these other types of graphs uninformative. In contrast, violin-plots with integrated boxplots are informative for results that are not described for the purpose of expressing similarities directly, because these plots nonetheless contain useful information about the type of distribution and a central tendency estimate (i.e., the median). More important, these plots are only useful when a few groups are compared. Superimposed graphs or violin-plots might be overloaded if there are too many groups compared (see Figure 6 for an example).

### Method

#### Participants

There were 291 participants (*M*_age_ = 33.75, *SD* = 11.17, 46% women) who remained in the analysis after excluding 24 participants because they failed an instruction check twice (Oppenheimer, Meyvis, & Davidenko, 2009), and 1 participant was excluded because he or she responded to all items with 0. Participants were recruited via a paid online platform.

#### Materials and procedure

**Figure 5:**
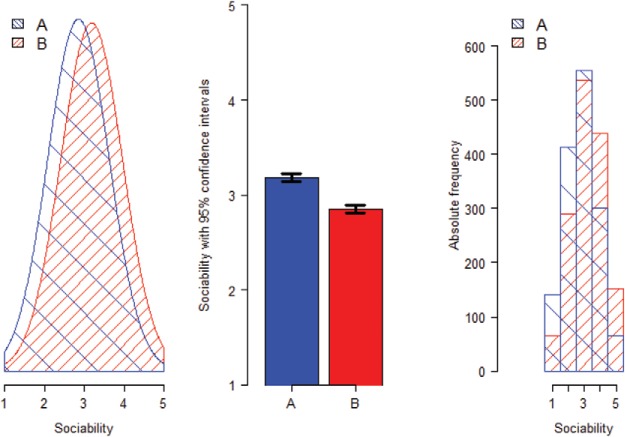
Three modes of depicting the same data: two superimposed normal distributions, a bar chart with 95% confidence intervals, and two superimposed histograms for *d* = .43 and PCR = 83 (percentage of common responses).

Three types of graphs were created, displaying simulated data with 1,500 participants in each group (i.e., the average sample size per country in the World Values Survey data). Data were simulated from a normal distribution, with a *SD* of 0.80 and an overall mean of 3. The three types of graphs are depicted in Figure 5: a graphical representation of the overlapping distributions assessed by the PCR measure, a default barplot with 95% CIs, and superimposed histograms that represent the PCS measure. Note that using a CI as error bars rather than *SE*s is a conservative measure, because bars representing *SE*s would have made the differences look larger. For each type of graph, nine versions were created with varying effect sizes: *d* = 0, 0.20, 0.40, 0.50, 0.60, 0.80, 1, 1.5, and 2. Participants were randomly allocated to rate one type of graph. The instructions for the participants were “In your opinion, to what extent do the data as depicted in this plot indicate that the two groups A and B are different or similar? Each group consists of around 1500 people.” To make the variable more concrete, we labeled it *sociability*. Participants responded on a slider measure, ranging from 0 (*very different*) to 100 (*very similar*). Also, for each graph, participants rated how comprehensible they found the figure on a 5-point scale ranging from 1 (*extremely incomprehensible*) to 5 (*extremely comprehensible*).^3^

Participants then ranked which of three types of statement (presented in random order) is the clearest and most informative way to describe scientific findings: “The difference between men and women was Cohen’s *d* = .43 with Cohen’s *d* being the difference in the two groups’ means divided by the average of their standard deviations,” “The overlap of the responses given by men and women was 83 percent,” and “83 percent of the responses given by men were mirrored by women.” Finally, participants responded to some demographic items, including their education level and their statistical training, before being debriefed and thanked.

### Results and Discussion

We first compared participants with a university degree and some or a lot of statistical training with the other participants, but obtained no significant interaction between graph type and educational level (*F*s(2, 283) < 2.04, *p*s > .13) or statistical expertise (*F*s(2, 283) < 1.57, *p*s > .18). Therefore, we report the results across educational levels and statistical training.

**Figure 6 fig6:**
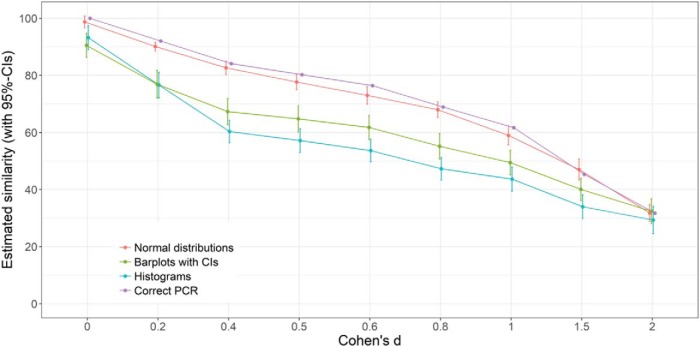
Estimated similarities for plots displaying data for various Cohen’s *d*s.

Next, we tested the influence of mode of presentation on perceived similarity. All but one of the nine between-subjects one-way ANOVAs reached statistical significance (see Table 1), indicating that mode of presentation had an impact on the perceived similarity between two groups. We observed a linear pattern, with the superimposed normal distribution plots leading to the most accurate perceptions, followed by the barplots, and the superimposed histograms (see Figure 6). In other words, people were more accurate at estimating similarities with the measure that represented the PCR than with any other measure. For example, for graphs displaying medium effect size (i.e., *d* = 0.50), the mean estimated amount of similarity was 78% for the superimposed normal distributions, 65% for the barplots displaying the means and CIs, and 57% for the superimposed histograms. The correct amount of overlap, using the PCR, is 80%. Thus, as expected, people underestimate similarities when shown results in the standard presentation format (means and CIs); they even tend to do so with the superimposed histograms, but presenting overlapping normal distributions attenuates the extent of this error. With respect to the comprehensibility ratings, the superimposed normal distributions were rated as more comprehensible (*M* = 3.92, median = 4, *SD* = 0.93) than the barplot condition (*M* = 3.59, median = 4, *SD* = 1.12, *p* = .03, PCR = 87, 95% CI [78, 98], AE = 0, 95% CI [0, 0], *d* = 0.32), and histogram condition (*M* = 3.50, *SD* = 1.13, *p* = .006, PCR = 84, 95% CI [73, 95], AE = 0, 95% CI [0, 0], *d* = 0.40).[Table-anchor tbl1][Fig-anchor fig6]

Finally, we examined participants’ ranking of the three methods as ways of presenting scientific findings. As a measure of effect size, we report the generalized η_G_^2^ ${\hat{\eta }}_{G}^{2}$, computed with the ez package in R (Lawrence, 2015), because of its comparability across many research designs (Olejnik & Algina, 2003). A within-subjects ANOVA revealed a significant effect of method, *F*(2, 510) = 306.55, *p* < .001, ${\hat{\eta }}_{G}^{2}$ = .55, with the PCS measure rated as the clearest and most informative way to present the findings (*M* = 1.47, *SD* = 0.59), followed by the PCR (*M* = 1.69, *SD* = 0.55), and then Cohen’s *d* (*M* = 2.84, *SD* = 0.52). Pairwise comparisons revealed that the responses to all three groups differed significantly from each other, *t*s(255) > 3.45, *p*s < .001. These results support our contention that the technical framing of Cohen’s *d* makes it less easy to comprehend, although we do not dispute its utility for capturing differences.

## Study 3

In Study 3, we replicated and extended Study 2 by using real data and testing the consequences of emphasizing either similarities or differences for attitudes toward the outgroup. Specifically, we presented 2,264 British and 1,615 Polish participants’ ratings of human values in one of three ways (superimposed normal distributions, bar-plots with a restricted *y*-axis, and bar-plot with unrestricted *y*-axis), which highlighted similarities or differences between the two groups (see Figure 4). We expected that highlighting similarities (superimposed normal distributions) would lead to more positive attitudes among British respondents toward Polish people, compared with highlighting differences (barplots with restricted *y*-axis). We expected the third condition to fall somewhere between the first two.

Polish people were chosen as an outgroup because they are the largest immigrant group living in the United Kingdom (Office for National Statistics, 2016) and around 25% of British people have less than favorable attitudes toward Polish people (British Future, 2014). The latter view is echoed by Polish people: 50% believe that people living in Britain have a negative attitude toward them (YouGov, 2014). In short, Polish people were selected because attitudes toward them vary quite substantially in our participant population.

We acknowledge that it was noted more than 60 years ago that restricting or truncating the *y*-axis makes an effect appear larger than it actually is (Huff, 1954). Nevertheless, based on our casual observations of journal articles, scientific posters, and presentations at conferences, bar- and line-graphs with restricted *y*-axes are still the predominant ways in which psychological findings are presented: Across all empirical articles published in 2017 in this journal, 41 used at least one bar- or line-graph with restricted *y*-axes to present their findings, while only 18 used unrestricted *y*-axes (χ^2^ = 8.97, *p* = .003).^4^ Further, the finding that most graphs do not provide any distributional information is in line with previous research, which found across two leading psychological journals that bar graphs were most common “and only about 10% of the graphs showed distributional information beyond central tendency” (Sándor & Lane, 2007; as cited in Lane & Sándor, 2009, p. 240). This dominant trend is not surprising given that a bar- or line-graph with a restricted *y*-axis is clearly effective at highlighting what most researchers are seeking: mean differences. However, whether or not this approach is teleological, it is important to test empirically whether there are potentially detrimental effects of using restricted *y*-axes graphs in comparison with other presentation formats.

### Method

#### Participants

Based on the results of Study 2, we assumed a medium effect size of *f* = 0.25. A power analysis revealed that a sample size of 58 participants per cell would be required for a power of .90. Before any data analysis, four participants who completed the survey in less than 45 s were excluded. Participants were recruited online and were compensated with £0.54, which equates to an hourly wage of £8.19 (average completion time was 237 s). The mean age of the remaining 251 participants was 37.30 (*SD* = 12.39, 62.65% women). There were 162 who had a university degree and 88 had less than a university degree. All participants were born in the United Kingdom, of British nationality, and had not participated in Study 2.

#### Design

A one-way design with three levels was used, with type of presentation (superimposed normal distribution, restricted bar-plot, and unrestricted bar-plot) as a between-subjects factor. Participants were randomly allocated to one of the three conditions.

#### Materials and procedure

**Figure 7 fig7:**
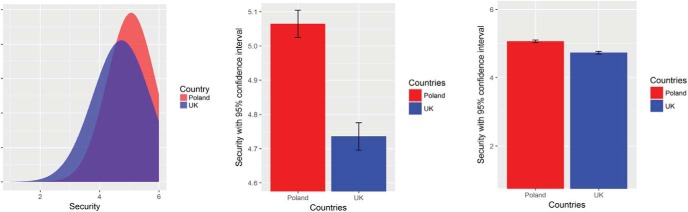
Three modes of depicting the same data: two superimposed normal distributions and two barplots (one with restricted range, the other with full range) with 95% confidence intervals for PCR = 86 (percentage of common responses), AE = 6 (absolute effect), and *d* = .36 (Study 3). Participants were exposed to 10 graphs simultaneously, one for each value.

Three types of graph were created, displaying the responses of 2,264 British and 1,615 Polish participants in the European Social Survey to Schwartz’s (1992) 10 values, which were measured with the 21-item version of the Portrait Value Questionnaire (Schwartz et al., 2001; 7th round; see Study 1 and the study reported in the online supplemental materials for more details about this questionnaire). For each of the 10 values we created one graph of each type (see Figure 7 for examples for the value type *security*). The average PCR across all 10 values was 88 (average AE = 6, average *d* = .31). All graphs of a given type were presented together (i.e., participants saw 10 graphs simultaneously). All participants were told that they “will be asked to rate the extent to which the graphical information displayed reflects differences and similarities between groups of people (e.g., British and Polish). All the figures we present are based on actual data from large European representative samples.” This was followed by a link to the Web page of the European Social Survey and an indication that all the presented data are real. Next, participants were more specifically informed that they would see “ratings of 10 human values in large representative samples from two countries. You will be shown 10 figures displaying the responses to one human value at a time, before being asked to respond to four questions.” The four items were the same in all three conditions. The first asked how similar the values of British and Polish people are; the second asked how easily British and Polish people can get along with each other; the third item was the inclusion of the other in the self-scale (IOS; Aron, Aron, & Smollan, 1992): “Which of the following 7 pairs of circles best describes the relationship between British people (self) and Polish people (other)?”; the fourth item asked for an overall evaluation of a typical Polish person. The first, second, and fourth items were answered on a slider scale ranging from 0 (*very different/not well at all/extremely unfavorable*) to 100 (*identical/perfectly well, extremely favorable*). The IOS-item displayed seven pairs of circles with a varying degree of overlap (from 0% to approximately 80%), with one pair to be selected by the participants. Because of a technical issue, the fourth item was only displayed to the last 151 participants.[Fig-anchor fig7]

### Results and Discussion

First, we tested whether education moderated any effect of graph type by including education (lower vs. higher) as a second factor in the four two-way ANOVAs. However, none of the four interactions (one for each item) were significant: *F*(2, 243) = 0.69, *p* = .50, *F*(2, 243) = 0.89, *p* = .42, *F*(2, 243) = 0.62, *p* = .54, and *F*(2, 243) = 1.53, *p* = .22, respectively (see Table 2 for the order of the items). This indicates that education does not moderate the results.[Table-anchor tbl2]

**Figure 8 fig8:**
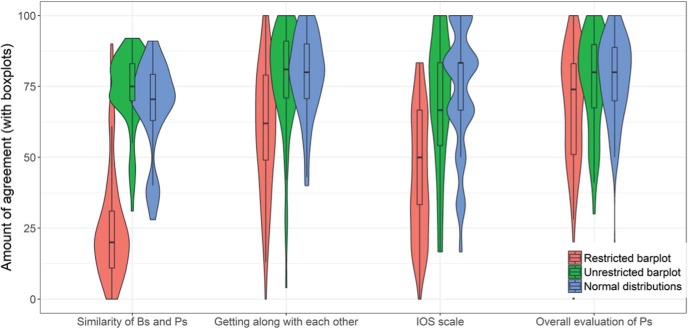
Amount of agreement to four items (Study 3). B = British people; P = Polish people; IOS = inclusion of the other in the self (broadened from the 1–7 response scale to 0–100).

Next, we performed four one-way ANOVAs. As shown in Table 2, all four ANOVAs were significant and post hoc tests revealed that the mean differences between the normal distribution and the restricted barplot conditions were in the expected direction, reflecting varying degrees of similarity between the two groups. The smallest amount of similarity was found for the perceived similarity of values held by British and Polish people. Participants who saw the values of British and Polish people as superimposed normal distributions also thought that the two groups get along more easily, are closer to each other, and that British people have a more positive evaluation of Polish people, compared with those who saw the restricted barplots (see Figure 8). As the PCR for the first item is below 50 and the AE above 50 (PCR = 24, 95% CI [13, 35], AE = 51, 95% CI [46, 53]), this is an example of real differences between groups, according to our taxonomy. Also, we found group differences between the participants who saw the barplots with unrestricted *y*-axes and those who saw them with restricted *y*-axes (PCR = 15, 95% CI [6, 25], AE = 55, 95% CI [49, 60]). Thus, the currently predominant way of presenting scientific results, with graphs using a restricted *y*-axis, led to an overestimation of the actual differences. Participants in the unrestricted barplot condition gave the same responses, on average, as participants in the normal distribution condition.[Fig-anchor fig8]

## Study 4

**Figure 9 fig9:**
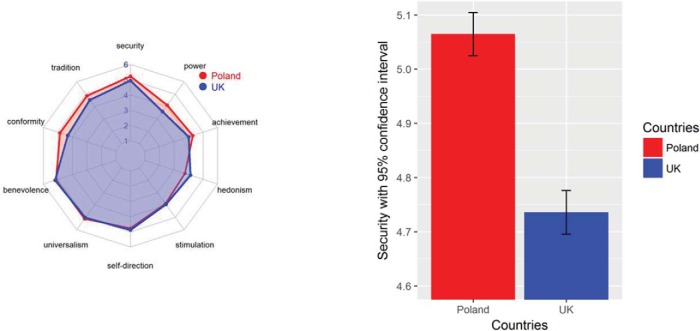
Two modes of depicting the same data: a radar-chart and a barplot with 95% confidence intervals for PCR = 86 (percentage of common responses), AE = 7 (absolute effect), *d* = 0.36. Note that 10 barplots were presented simultaneously, one plot for each value type (Study 4).

In this study, we tested whether the principal findings of Study 3 would also hold in high school students, with a different type of graph that highlights similarity: a radar-chart (see Figure 9). The data again concern similarities and differences in human values. More specifically, we examined whether the currently predominant way of displaying results (restricted barplots with error bars) would again lead to an overestimation of differences, whereas presenting the same data in the form of a radar-chart would lead to a more accurate perception of the results. We also tested whether the different modes in presentation would have an impact on participants’ attitudes toward an outgroup.[Fig-anchor fig9]

### Method

#### Participants

Based on the results of Studies 2 and 3, we assumed a large effect size of *d* = 0.80. A power analysis revealed that a sample size of 28 participants per cell would be required for a power of .90. Participants were 54 high school students (*M*_age_ = 17.13, *SD* = 0.73, 81.48% women).

#### Materials and procedure

Participants were presented with either a radar-chart displaying all 10 values or 10 single barplots with CIs (see Figure 9). Subsequently, participants responded to three items on a scale from 0 to 100, similar to Study 3: “How similar are the values of British and Polish people?”, “How easily do you think British and Polish people can get along with each other?”, and “How much do you think British and Polish people like each other?” Again, participants were informed that these results came from real data provided by 2,264 British respondents, originating from www.europeansocialsurvey.org.

### Results and Discussion

**Figure 10 fig10:**
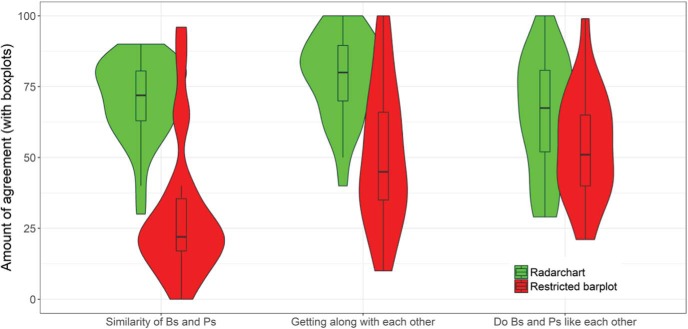
Amount of agreement with three items (Study 4). B = British people; P = Polish people.

Participants who were exposed to the information format emphasizing similarity (i.e., radar-chart) subsequently exhibited more intergroup positivity on all three dependent variables than did participants exposed to the traditional format emphasizing differences (i.e., barplots). That is, participants perceived the values of British and Polish people to be more similar (*M* = 70.45, *SD* = 14.23) than did those in the difference condition (*M* = 30.61, *SD* = 24.70), *t*(32.71) = 6.93, *p* < .001, PCR = 30, 95% CI [7, 53], AE = 50, 95% CI [39, 58], *d* = 2.06. Participants in the similarity condition also thought that British and Polish people get along with each other more easily (*M* = 78.65, *SD* = 14.97) than did those in the difference condition (*M* = 50.43, *SD* = 24.51), *t*(33.95) = 4.89, *p* < .001, PCR = 47, 95% CI [25, 68], AE = 35, 95% CI [18, 46], *d* = 1.44. Finally, participants in the similarity condition thought that British and Polish people like each other more (*M* = 65.30, *SD* = 20.43) than did those in the difference condition (*M* = 54.29, *SD* = 19.17), *t*(44.88) = 1.97, *p* = .056, PCR = 78, 95% CI [55, 98], AE = 17, 95% CI [0, 31], *d* = 0.55 (see Figure 10). Overall, the currently predominant way of presenting scientific results on graphs with a restricted *y*-axis led to an overestimation of the actual differences between the two groups.[Fig-anchor fig10]

## Study 5

In Studies 2 to 4 we relied on simulated or archival data. Further, the similarities in the presented data were substantial. In Study 5, we aimed to replicate and extend the results of Studies 2 to 4 by using data from two large, influential, and controversial studies. The criteria for selecting influential studies were that the study had to be (a) disseminated to the general public (e.g., through news coverage), (b) based on a large sample size, (c) focused on the comparison of two polarized groups, and (d) controversial. The first selected study involved comparisons of the five moral foundations of conservatives and liberals (Graham et al., 2011). The second study was a meta-analysis in which it was found that the IQ of religious nonbelievers is around seven points higher than that of religious believers (Zuckerman, Silberman, & Hall, 2013). We again presented the key findings of both studies either with a restricted barplot or with superimposed normal distributions. Additionally, we presented the five moral foundations in the form of a simple line-graph—one of the standard ways in which Graham, Haidt, and colleagues present their findings both in scientific articles (e.g., Graham, Haidt, & Nosek, 2009) and in the dissemination of their findings to the general public (e.g., see their Web page at yourmorals.org or Haidt’s TED-Ed talk https://www.youtube.com/watch?v=8SOQduoLgRw). Finally, we explored whether the mode of presentation would also affect participants’ ratings of the quality of the research.

### Method

#### Participants

Based on the results of Studies 3 and 4, we assumed a large effect size of *f* = 0.40. A power analysis revealed that a sample size of 27 participants for each of the three cells would be required for a power of .90. In total, 157 participants were recruited online with a mean age of 33.37 years (*SD* = 11.44, 43.87% women). There were 115 participants who had a university degree and 40 had less than a university degree (two missing values). All participants were living in the United States and none had participated in Studies 2 or 3.

#### Materials and procedure

Participants were informed that they would see “the findings of two influential scientific studies. You will be asked a few questions about the findings themselves and about your impression of the quality of the scientific studies.” Participants were then randomly allocated to one of the three types of graphs displaying the moral foundations of conservatives and liberals and answered six questions after being given a short introduction to and definition of the moral foundations. Next, participants were randomly allocated to one of two groups and shown the results of the IQ comparisons of religious believers and nonbelievers, and answered the same six questions again. Finally, participants reported the strength of their religiosity on a 7-point scale ranging from 1 (*not religious at all*) to 7 (*very religious*), their religious denomination (if any), their political orientation on a 11-point scale ranging from 1 (*very left*) to 11 (*very right*), and how they estimate their intelligence compared with everyone living in the United States, ranging from 0 (*far below average*) to 100 (*far above average*).

We used the means and *SD*s of all five moral foundations for 4,128 conservatives and 21,933 liberals reported by Graham et al. (2011) to create the graphs we used as stimuli material (see online supplemental materials). The amount of similarity between conservatives and liberals was substantially smaller than was the case for the value comparisons used in Studies 3 and 4. The PCR ranged from 67 (*d* = 0.86) for *harm* to 37 (*d* = 1.81) for *purity*, with liberals scoring higher on harm and conservatives higher on purity. The layout of the graphs with the superimposed normal distributions and the barplot was the same as the one used in Study 3 (see Figure 7, left and middle panel; see the online supplemental materials for the graphs). We created one graph for each of the five moral foundations and presented all five graphs together. The line-graph contained all five moral foundations together on the *x*-axis, with two lines representing the mean relevance for conservatives and liberals, respectively.

Participants in all three groups were informed that they would “see the results of a study examining how more than 25,000 conservatives and liberals from several countries (but mainly the US) rated the importance of five moral concerns for them personally.” This was followed by a short summary of the moral foundations research, including the method and definitions of each of the moral foundations. Additionally, we added a link to the full version of the Graham et al. (2011) article. Next, participants responded to six items on a slider measure ranging from 0 to 100 with appropriate labels for the endpoints: (a) “How similar are the moral foundations of conservatives and liberals?”, (b) “How easily do you think conservatives and liberals can get along with each other?”, (c) “Do the findings of this study make sense to you overall?”, (d) “What is your overall impression of the results of this study?”, (e) “As you know, scientific evidence can vary in quality. How do you judge the quality of this study?”, and (f) “Do you think the researchers who conducted the study were biased (i.e., were hoping to get a specific finding), making the results less valid?”

The data for the graphs displaying the IQ difference of religious believers and nonbelievers were extracted from Zuckerman et al. (2013), who in their meta-analysis found that the mean IQ difference is 7.3 points. Because more exact descriptive statistics were missing, we used the standard distribution of the IQ (*M* = 100 and *SD* = 15) to create the graphs. Specifically, we assumed that the IQ of the believers to be 100–7.3/2 = 96.35 and the IQ of nonbelievers to be 100 + 7.3/2 = 103.65. This means that the amount of similarity between the two groups is PCR = 81 (*d* = 0.49). We further assumed that the overall sample size was 10,000, a rather conservative estimate given that the meta-analysis contained 63 studies (determining the exact number per cell is not possible, because many studies were correlational). The design of the graph with the superimposed normal distribution and the graph with the barplots was again the same as in Study 3 (see Figure 7, left and middle panel; the only change was that the colors were orange and green; see the online supplemental materials for the graphs). This study was introduced and summarized in a similar way to the one about the moral foundations; the six items were adapted to the content of the study (e.g., “How similar is the IQ of believers and non-believers?”).

### Results and Discussion

First, we tested whether education moderated any effect of graph type by including education (lower vs. higher) as a second factor in the 12 two-way ANOVAs. However, none of the six interactions of education with the six moral foundations items was significant (*F*s < 1.20, *p*s > .30) and only one of interactions of education with the six IQ items was significant (*F*s < 1, *p*s > .50 for the other five items). Therefore, we collapsed the responses of lower and higher educated people. We then tested whether political attitude moderated the effect for the graphs presenting the moral foundations of conservatives and liberals, and whether strength of religious belief moderated the effect for the graphs presenting the IQs of believers and nonbelievers. We did this in a series of linear regressions including interaction terms. However, out of the 12 interaction terms we computed for the moral foundation analysis (barplots vs. line-graphs and barplots vs. normal distributions for all six DVs), only one was significant, *B* = 2.67, *SE* = 1.35, *t*(147) = 1.98, *p* = .049, while the other 11 interaction terms were not significant, *t*s(146–149) < 1.64, *p*s > .10. For the six interactions of the IQ portion of the study, again only one was significant, *B* = −4.43, *SE* = 2.08, *t*(149) = −2.13, *p* = .035, whereas the other five were not, *t*s(149–150) < 1.78, *p*s > .07. Therefore, we collapsed our results across political attitudes and strength of religious belief.

In a next step, we tested whether response time moderated any of the observed effects. This assumption is based on the elaboration likelihood model (Petty & Cacioppo, 1986), which leads to the prediction that people who respond faster might be more influenced by shallow attributes (peripheral route), whereas those who spend more time elaborating (central route) would not. Specifically, we assumed that the effect of presentation mode would be stronger for those who responded faster because these participants spent less time carefully examining the graphs and the descriptions. However, none of the 12 interaction terms was significant (all *F*s < 1.50, all *p*s > .20).

**Figure 11 fig11:**
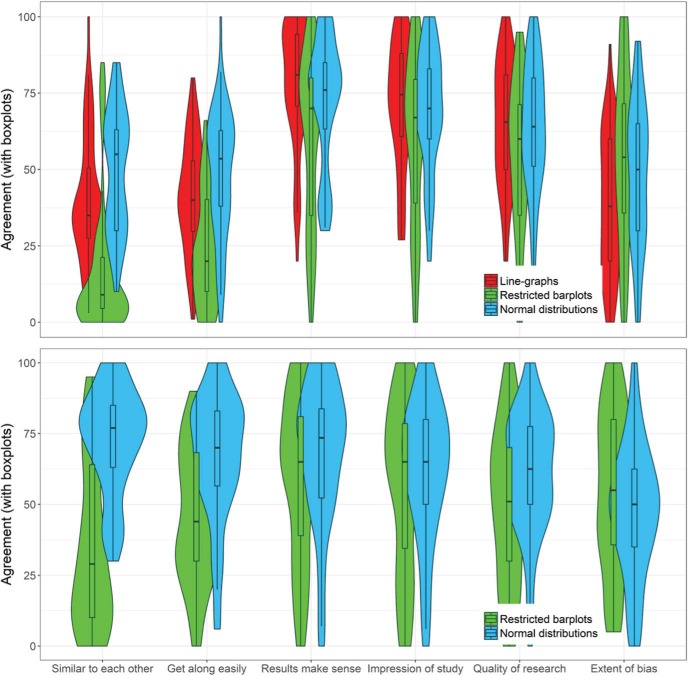
Amount of agreement with all items after being exposed to different types of graphs displaying moral foundations of conservatives and liberals (top panel) and the IQs of believers and nonbelievers (bottom panel; Study 5).

As predicted, and consistent with the findings of Studies 3 and 4, participants clearly considered the similarities to be larger between conservatives and liberals and between believers and nonbelievers when the findings were presented in the form of superimposed normal distributions as opposed to barplots with a restricted *y*-axis (Table 3 and Figure 11). Additionally, participants in the normal distribution condition thought that members of both groups could get along easier with each other than participants in the barplot condition. When the findings were presented in the form of a line-graph, the perceived similarities between the two groups and the perceived ease with which they could get along with each other fell between the other two conditions. Additionally, we found that the overall quality of both studies was perceived to be higher by those participants who saw the superimposed normal distributions. No consistent pattern was found for the other three quality-related items, showing that the mode of presentation did not affect, for example, the extent to which the researchers were perceived to be biased.[Table-anchor tbl3][Fig-anchor fig11]

## General Discussion

The five studies presented here found evidence consistent with our argument that similarities between groups should be reported alongside mean differences. This argument was supported by three findings: similarities are typically larger than mean differences; reporting similarities leads on average to more accurate interpretations of research findings; and reporting similarities between ingroups and outgroups leads to more positive attitudes toward the outgroups.

Rather than focusing on comparisons of means, as is routinely done in psychological research and other social and medical sciences (e.g., when reporting *t* tests), we argue that it is important to analyze and interpret the extent of overlap between all responses. Doing this enables recipients of scientific communications to draw different conclusions from the data and also has some intriguing implications, which we discuss below, after summarizing the findings. Study 1 showed that across all 22 variables we examined, the average PCR was 93 when we compared people from different genders, ages, educational attainments, incomes, countries, and religious denominations. This high level of similarity was corroborated by the AE. With an average of .05 across all categories and variables, this shows that the median difference between any two groups was only one-twentieth of the possible difference on the scale in question. Indeed, the average of the largest mean differences between two groups across two studies (Study 1 and a similar study of a different dataset, containing responses from 29 European countries [*N* = 54,082, reported in the online supplemental materials]) and all categories on all variables is AE = .16 (median = 9), which is less than one-seventh of the possible range (mean of the 132 smallest PCRs = 78 and median = 86 across all variables and categories).

Countries were on average less similar to each other than were groups within other categories. This challenges the claim that countries are more similar to each other than are people with different socioeconomic status (Greenfield, 2014). The lowest degree of similarity we found across all comparisons was for moral attitudes toward personal and sexual issues. This result is broadly consistent with Graham et al.’s (2011) evidence that the largest differences between various groups of people—mainly liberals and conservatives—is on the moral dimension *purity*, which is closely related to moral attitudes toward personal and sexual issues.

In summary, the findings of Study 1 and the study reported in the online supplemental materials support the universalism claim in cross-cultural research, which holds that values, attitudes, and beliefs are only weakly influenced by cultural factors (Berry et al., 2011). However, we can now be more confident that country has a relatively strong influence on variables like human values, independently of religious denomination, education and income level, or age distribution, extending previous research on cross-country similarities of values (Fischer & Schwartz, 2011; Schwartz & Bardi, 2001). If country differences were mainly because of differences in religious denomination, education level, or age distribution, we would have found groups in these categories to be less similar to each other than countries. By contrast, we found that countries were less similar to each other than were groups in these other categories.

The finding that similarities between various groups outweigh differences across a range of psychological variables might be considered as surprising giving that most research claims to have found differences between groups (Fanelli, 2010). However, from a biological or evolutionary point of view this is less surprising: Well over 99% of our DNA is shared and every group or society needs to develop some shared standards of communication and action to function effectively. For example, it has been argued that social selection “favored genes that gave rise to new, more pro-social motives” (Boyd & Richerson, 2009, p. 3281). More specifically, as Schwartz and Bardi (2001) have argued, benevolence values (e.g., loyalty, honesty) are similar across countries because they guide human cooperation and supportive relationships. Universalism values (e.g., equality, broad-mindedness) are important in every society because they guide the commitment to outgroup members, with whom most groups and societies need to form some kind of relations either in schools or workplaces or, on a larger setting, when trading. Security is also highly valued across countries because in evolutionary terms there have often been threats from out- and ingroup members. The resulting similarities are manifested in many international agreements. For example, more than 190 countries belong to international crime police organizations (ICPO or INTERPOL), which indicates that they share at least some common policies regarding prevention of and responses to criminal behavior. Another example is the Universal Declaration of Human Rights (1948), which has been signed by almost every nation and demonstrates that humans share a common system of values (Bobbio, 1996). Below we first discuss some implications of our findings for the reporting of research findings, before discussing an example of these implications, focusing on cross-cultural research and the definition of culture.

### Implications

Given the focus of the principal statistical tests used in psychology, it is common for researchers to focus almost exclusively on mean differences. This article provides several methods for also calculating and displaying similarities. Reporting similarities helps readers to interpret mean differences more appropriately. Consider an example taken directly from the results of the between-country comparisons: A researcher comparing two countries could report that the mean difference between them had an effect size of *d* = .39, but with 84% common responses and only an 11% difference in scale use. Reporting the results in this way avoids the tendency to oversimplify the findings by focusing simply on mean differences. Focusing only on mean differences is one way in which social scientists might inadvertently steer people into regarding differences between groups as entrenched. As Studies 3 to 5 demonstrate, reporting observed similarities between groups offers a way of counteracting racism, xenophobia, and prejudice regarding people from another country or people who hold another ideological view. Therefore, we argue that, especially in the case of research that focuses on comparisons between polarized groups (e.g., those based on ideology, religion, or education), researchers should not fail to highlight similarities between the groups, especially when communicating to the public (e.g., via press releases). More important, in contrast to previous research that has found that highlighting similarities improves intergroup attitudes (e.g., Brown & Abrams, 1986), in the current research we have presented exactly the same information in different ways, rather than relying on different information to manipulate a focus on similarity or difference. Further, our approach allows presentation of information about large groups of participants, preventing group subtyping, which is a psychological response often used to protect stereotypes (Richards & Hewstone, 2001).

Furthermore, the presentation of similarity information is useful even when differences are small but reliable. Although people might infer that similarity is high from the fact that the differences are small, this inference runs the risk of being misleading if it is not concretely framed. Discrimination on the grounds of ethnicity or gender is a case in point. For example, if people belonging to a specific group (e.g., ethnic minorities or women) earn less than those in other groups, the similarity measures discussed here would enable researchers to frame such differences more concretely. For example, it is less easy to comprehend a statement that the (small) gender wage difference within a specific company is *d* = 0.24 than a statement that 90% of men and women employees share the same salary, but that in most of the remaining cases women have lower salaries. This concrete interpretation fosters greater realization that the key problem is to identify when women’s salaries are lower and to understand why this is the case. Thus, the meaning and implications of small effects are likely to be *enhanced* when researchers report these effects in the context of the observed similarities (Prentice & Miller, 1992).

A stronger focus on similarities in published research should also help to reduce the “file-drawer” problem (Rosenthal, 1979) because both statistically large and statistically small differences between groups are potentially more interesting against the backdrop of similarity information. This would make the documentation of similarities between some groups and variables an interesting exercise in its own right, which might also increase the number of variables that are used to compare groups. At the moment, variables are often chosen on the basis of their perceived likelihood of revealing differences, potentially biasing the literature before any data are collected (Fiedler, 2011).

It should be noted that our proposal is descriptive. Null hypothesis significance testing and Bayesian statistics are complementary with our approach. When comparing two groups, larger *p* values and smaller Bayes factors—*ceteris paribus*—imply greater similarity. Crucially, however, neither of the two indices nor any of the commonly used effect sizes directly *communicates* similarity. We suggest that as well as reporting differences, researchers should report measures of similarity to fully reflect the nature of the effects in question.

### Reanalyzing the Concept of Culture

Culture is the construct most often used in cross-cultural research. It has been defined in various ways, for example by Hofstede (2001) as the “the collective programming of the mind that distinguishes the members of one group or category of people from another” (p. 9). Culture further determines “the uniqueness of a human group in the same way personality determines the uniqueness of an individual” (p. 10). This definition of Hofstede has been challenged, because the assumption that there exist both “substantial within-group agreement and between-group differences” (Schwartz, 2014, p. 6) has been empirically rebutted. For both personality traits and values, within-country variability has been found to be 9 to 15 times larger than between-country variability (Allik, 2005; Berry et al., 2011; Fischer & Schwartz, 2011). Based on these findings, it has been argued that larger differences can be found between groups with a different socioeconomic background (Greenfield, 2014) or across variables other than values (Morris, 2014).

However, both Greenfield (2014) and Morris (2014)—as well as most other researchers who claimed to have found large differences across countries—based their conclusions on comparisons between means and the rules of thumb proposed by Cohen (1992) concerning when an effect size should be called small, medium, or large. As we demonstrated in the above, equating mean differences with group differences is mostly unjustified because even “highly significant” differences between means usually involve large similarities between the groups in question. In fact, the average similarity found in Study 1 and the study reported in the online supplemental materials was above 90% across various categories and variables. Nevertheless, we also found some differences between countries for variables such as moral attitudes toward personal-sexual issues. Further, as we argue in the limitation section below, studying other variables or participant groups might have resulted in smaller similarities.

Based on these findings and reflections, we propose a more flexible, relativistic, and quantitative view of culture: Individuals or groups of individuals belong to different cultures if they are more different than similar on a specific variable (e.g., if the PCR is below 50). If all or most members of two groups differ from each other on a specific variable, they arguably belong to different cultures. For example, in Study 1, we found that Pakistanis, on the one hand, and Dutch and Swedish participants, on the other, displayed differences in their moral attitudes toward personal-sexual issues such as abortion or homosexuality. However, for many other variables (cf. Supplementary Table 1), the similarities between these groups were again larger than the differences. This means that the majority of Pakistani and Dutch/Swedish participants belong to different cultures, but only with regard of moral attitudes toward personal-sexual issues. For many other variables (e.g., values), they are culturally more similar than different and, therefore, do not belong to different cultures.

In a similar manner, the United Kingdom and most continental European countries belong to both similar and different cultures. They belong to different cultures with regard to the side of the road on which people drive, currency, language, and the size of university tuition fees. However, with regard to many other societal, political, and psychological variables, they belong to the same culture. To phrase it more broadly: If two groups of people differ along variable X, they belong to two cultures on X. There is little empirical support for the default approach in cross-cultural research, which is to ascribe people living in one country a lot of attributes simply because this country differs from other countries in terms of mean scores on one abstract dimension. This might explain why a range of studies have failed to find even mean differences between so-called individualistic and collectivistic countries, despite the fact that differences were or could have been expected based on differences in the individualism-collectivism dimension (e.g., Berry et al., 2011; Fiske, 2002; Georgas, Berry, van de Vijver, Kagitçibasi, & Poortinga, 2006; Lee & Johnson-Laird, 2006; Nisbett, 2004). In other words, people should be simultaneously distinguished directly on the putative cultural categories to which they are assigned and the related psychological variables, rather than using demographic categories alone (e.g., country of origin) to infer psychological differences.

This view of culture as variable-dependent can stimulate more empirically driven research. For example, the large similarities found between Eastern and Western countries on individualism (e.g., self-direction) and collectivism (e.g., tradition values) also indicate that approximately half of the participants in a Western country (e.g., United States) score higher on individualism than half of the participants of an Eastern country (e.g., Japan)—*and* vice versa. A large cluster-analysis could be used to explore what distinguishes those scoring high on individualism from those scoring low on it (e.g., type of profession). This could also be done across many categories and variables to find groups of people who differ on one or more variables.

### Limitations

Although our results show that similarities are much larger than differences over a wide range of measures, they are likely to be dependent on the measures used. The variables included in our studies are widely used in social sciences to examine differences between groups, but we might have detected smaller similarities with other measures (cf. Morris, 2014). For example, although questions about whether it is appropriate to make jokes about a religion or to burn its holy scriptures are likely give rise to similar responses among many if not all groups of people, cultural and religious similarities might well be smaller if respondents were asked whether such behavior would be a reason to enact corporal or capital punishment. Indeed, our data indicate that personal moral issues exhibit relatively low levels of between-country similarity. Thus, more measures focused on such issues might have revealed lower levels of similarity, on average.

Another limitation concerns the sample used. Although our samples from the WVS and the European Social Survey (ESS; see online supplemental materials) can be considered as representative (Fischer & Schwartz, 2011), the respondents are mostly literate, willing to complete a questionnaire, and may be more likely to be influenced by other cultures because they were willing to participate in a cross-country survey (polyculturalism; Morris, Chiu, & Liu, 2015). Indeed, other research has shown that differences between small-scale societies such as savanna foragers in Tanzania and tundra-taiga hunters and fishers in Siberia are larger than those between student samples in different countries (Henrich et al., 2006).

To reiterate a point made earlier in this article, we are not arguing that small mean differences are unimportant. In many instances, small to large mean differences *are* meaningful, despite partially reflecting large similarities (cf. Prentice & Miller, 1992). Take the similarities in values across countries, for example. In Study 1 and the study reported in the online supplemental materials, we found that the average similarities in values exceed 90% across more than 70 countries, indicating that the mean differences are small. Nevertheless, the country means for some value types, such as self-direction or autonomy, are strongly (*r*s > .60) correlated with how peaceful or democratic a country is (Basabe & Valencia, 2007; Schwartz, 2006). In such instances, it can be useful to report the degree of similarity because this shows the importance of relatively small deviations from a perfect degree of similarity. The ability of small differences to matter greatly is not in doubt; just as tiny differences in DNA between people can have enormous impact on their lives, small differences in psychological variables can have repercussions that extend well beyond their apparent size.

All that we suggest is that research should complement the dominant focus on differences with an explicit recognition of similarities; otherwise these similarities go unnoticed. That said, future research might identify other ways to go about this task. For instance, one limitation of our recommended indices pertains to the restriction of the AE to variables that have noninfinite lower and upper bounds (e.g., Likert or bipolar scales). Although the AE can be generalized to other scales, such as RTs, researchers might discover easy-to-use alternatives that work across virtually infinite ranges. For now, all that is required is to define a reasonable minimum and maximum within which a specific proportion (e.g., 99%) of participants fall across various groups of participants and tasks.

### Conclusion

The present research demonstrates alternative ways of reporting quantitative data comparisons between groups. In the course of making more than 168,000 comparisons, we found that similarities between any two groups of humans generally far outweigh the differences between them. Although the degree of similarity depends on the groups and measures used, the fact that our conclusions are based on a large range and number of comparisons leads us to expect that future research will also find substantial similarity on many psychological variables.

## Supplementary Material

10.1037/pspi0000154.supp

## Figures and Tables

**Table 1 tbl1:** Effects on Perceived Similarity in Nine One-Way Between-Subject ANOVAs and Pairwise Comparisons (Study 2)

Cohen’s *d* of graph	Normal *M* (*SD*)	Barplot *M* (*SD*)	Histogram *M* (*SD*)	*F*	Pairwise comparisons with PCR [95% CI]/*d*			B-H
					${\hat{\eta }}_{G}^{2}$	N-B	N-H	
0	98.76 (10.20)	90.50 (20.61)	93.24 (21.09)	4.46*	.02	80 [70, 91]/0.51***	87 [79, 97]/0.33*	95 [83, 100]/−0.13
.2	90.12 (7.50)	76.93 (23.52)	76.62 (22.62)	23.34***	.07	70 [62, 78]/0.76***	69 [62, 74]/0.80***	99 [87, 100]/0.01
.4	82.65 (11.71)	67.31 (22.60)	60.34 (19.95)	69.75***	.18	67 [57, 76]/0.86***	50 [40, 59]/1.36***	87 [75, 98]/0.33*
.5	77.68 (13.49)	64.77 (22.72)	57.18 (21.10)	54.24***	.16	73 [63, 83]/0.69***	56 [46, 66]/1.15***	86 [74, 97]/0.35*
.6	73.01 (15.19)	61.78 (20.68)	53.65 (19.92)	52.55***	.15	76 [64, 87]/0.62***	59 [47, 70]/1.09***	84 [72, 95]/0.40**
.8	67.97 (13.61)	55.19 (21.84)	47.29 (20.08)	59.27***	.17	72 [62, 83]/0.70***	55 [44, 65]/1.20***	85 [73, 96]/0.38**
1	58.97 (16.63)	49.47 (20.98)	43.65 (21.39)	29.47***	.09	80 [69, 91]/0.50***	69 [57, 80]/0.80***	89 [77, 99]/0.27
1.5	47.01 (18.25)	40.05 (18.99)	34.00 (20.79)	22.22***	.07	85 [74, 96]/0.37*	74 [62, 85]/0.66***	88 [76, 99]/0.30*
2	31.79 (14.58)	32.39 (20.94)	29.33 (23.99)	.74	.00	99 [86, 100]/−0.03	95 [83, 100]/0.12	95 [83, 100]/0.14
*Note*. CI = confidence interval; PCR = percentage of common responses; ANOVA = analysis of variance; AE = absolute effect; Normal (N) = superimposed normal distributions; barplots (B) = restricted bar-plots; N-B-H: pairwise comparisons with PCR [95% CI] and Cohen’s *d*. AEs are omitted for brevity. ANOVA *df*s 1/289.								
* *p* < .05. ** *p* < .01. *** *p* < .001.								

**Table 2 tbl2:** Analyses of Group Ratings in Four One-Way ANOVAs and Post Hoc Tests (Study 3)

Dependent variables	Normal *M* (*SD*)	Restricted *M* (*SD*)	Unrestricted *M* (*SD*)	ANOVAs			Pairwise comparisons with PCR [95% CI]/*d*		
				*F*	*df*s	${\hat{\eta }}_{G}^{2}$	N-R	N-U	R-U
Similarity of B and *p* values	67.75 (16.04)	25.66 (19.69)	73.91 (12.59)	214.68***	2, 247	.63	24 [13, 35]/2.34***	83 [72, 95]/0.43*	15 [6, 26]/2.91***
How easy do B and P get along?	79.01 (14.13)	59.99 (22.34)	77.67 (18.57)	27.25***	2, 247	.18	61 [50, 72]/1.02***	97 [85, 100]/0.08	67 [54, 79]/0.86***
IOS scale	5.42 (1.30)	3.65 (1.32)	5.20 (1.39)	43.84***	2, 247	.26	50 [38, 61]/1.35***	93 [81, 100]/0.17	57 [44, 69]/1.14***
Overall B evaluation of Ps	76.92 (16.43)	68.29 (20.72)	76.64 (16.85)	3.71*	2, 148	.05	82 [66, 97]/0.46*	99 [82, 100]/0.02	83 [68, 97]/0.44*
*Note*. CI = confidence interval; PCR = percentage of common responses; ANOVA = analysis of variance; AE = absolute effect; B = British people; P = Polish people; IOS = inclusion of the other in the self scale (Aron, Aron, & Smollan, 1992); Restricted (R) = restricted bar-plots; normal (N) = superimposed normal distributions; unrestricted (U) = unrestricted bar-plots; N-R = pairwise comparison of normal and restricted condition with PCR and Cohen’s *d*. AEs are omitted for brevity.									
* *p* < .05. ** *p* < .01. *** *p* < .001.									

**Table 3 tbl3:** Results of 12 One-Way ANOVAs and Pairwise Comparisons

Dependent variables	Normal *M* (*SD*)	Barplots *M* (*SD*)	Line-graph *M* (*SD*)	ANOVA		Pairwise comparisons with PCR [95% CI]/*d*		
				*F*	${\hat{\eta }}_{G}^{2}$	N-B	N-L	B-L
Moral foundations								
How similar are A and B	49.36 (19.54)	16.67 (21.66)	39.50 (20.90)	34.31***	.31	43 [24, 59]/1.59***	81 [64, 96]/0.49*	59 [41, 75]/1.07***
Ease of getting along	48.77 (20.88)	24.67 (18.68)	40.37 (17.73)	21.22***	.22	54 [39, 69]/1.22***	83 [67, 97]/0.43*	67 [51, 82]/0.86***
Do findings make sense?	72.27 (20.79)	61.02 (27.09)	78.25 (20.20)	7.52***	.09	82 [67, 96]/0.47*	88 [73, 99]/−0.29	72 [57, 86]/0.72***
Overall impression?	69.08 (20.20)	59.33 (27.70)	71.60 (20.25)	4.10*	.05	84 [69, 98]/0.40*	95 [78, 100]/−0.12	80 [66, 95]/0.51*
Quality of study?	64.06 (20.36)	53.54 (24.91)	64.92 (20.20)	4.36*	.05	82 [67, 96]/0.46*	98 [82, 100]/−0.04	80 [66, 95]/0.50*
Were researchers biased?	48.02 (23.21)	54.13 (28.02)	39.18 (22.81)	4.73*	.06	91 [75, 100]/−0.24	85 [69, 98]/0.38	77 [62, 92]/0.58**
Intelligence quotient								
How similar are A and B	72.54 (18.35)	36.53 (29.77)		83.17***	.35	47 [34, 59]/1.46***		
Ease of getting along	66.96 (22.28)	47.11 (23.67)		28.94***	.16	67 [54, 79]/0.86***		
Do findings make sense?	68.10 (24.52)	60.60 (27.87)		3.17	.02	89 [76, 99]/0.29		
Overall impression?	61.83 (24.48)	57.89 (29.30)		.83	.01	94 [82, 100]/0.15		
Quality of study?	60.86 (21.62)	50.74 (26.60)		6.84**	.04	83 [71, 96]/0.42**		
Were researchers biased?	47.56 (23.10)	54.64 (27.61)		3.01	.02	89 [76, 99]/−0.28		
*Note*. CI = confidence interval; PCR = percentage of common responses; ANOVA = analysis of variance; AE = absolute effect; Moral foundations = conservatives and liberals are compared; intelligent quotient = religious and nonreligious people are compared; barplots (B) = restricted bar-plots; normal (N) = superimposed normal distributions; line-graph (L); N-B, N-L, and B-L = pairwise comparisons between the three conditions with PCR and Cohen’s *d*. AEs are omitted for brevity. ANOVA *df*s for MF: 2/152–154, *df*s for IQ: 1/153–154.								
* *p* < .05. ** *p* < .01. *** *p* < .001.								

## Footnotes

1 The confidence intervals (CIs) for the absolute effect were bootstrapped based on 10,000 samples (see R code online). The CIs might not be symmetrically around the absolute effect (AE) because the distribution of the bootstrapped AEs is often skewed. The CIs for the percentage of common responses (PCR) were also bootstrapped.

2 We consider our newly developed measure, the percentage of common scores (PCS), to be as valid as the percentage of common responses (PCR), which is why we have also reported the results of the PCS in the online supplemental materials. However, we recommend the PCR because it is easier to compute, for example with this short command in R: 2 × pnorm((-abs(d))/2) × 100, and because people judge it more accurately (see Study 2).

3 We additionally asked participants to rank which of the three graphs they found easier to read and which one more informative using a within-subject design (participants saw Figure 5). However, the results were mixed. A within-subjects analysis of variance (ANOVA) for easier-to-read was statistically significant, *F*(2, 538) = 174.98, *p* < .001, with barplots ranked as the easiest-to-read graphs (*M* = 1.37, *SD* = 0.62), followed by superimposed normal distributions (*M* = 2.00, *SD* = 0.69), and superimposed histograms (*M* = 2.63, *SD* = 0.59). The within-subjects design allows one to compute common-language effect sizes: Two-hundred participants found the barplots easier to read than the superimposed normal distributions whereas 70 participants found the normal distributions easier to read. There were 199 participants who found the superimposed normal distributions easier to read than the superimposed histograms, whereas 71 found the superimposed histograms easier to read. In contrast, there was no statistically significant difference between the informativeness of the graphs, *F*(2, 524) = 0.28, *p* = .76.

4 We coded the type of graphs in all articles published in the *Journal of Personality and Social Psychology* in 2017 (Volumes 112 and 113). The majority of articles, 41, used bar- or line-graphs with (an at least somewhat) restricted *y*-axis and only two of them mentioned in the Figure caption the full range of the scale. Eighteen articles used unrestricted *y*-axes, 12 articles both type of visual presentations, 16 articles used another type of graph (e.g., scatterplot, mediational model, and forest plot), and 8 articles used no graphs with data in the main text.
