# Supplementary material for: A New Way to Look at the Data: Similarities Between Groups of People Are Large and Important
Source: J Pers Soc Psychol. 2018 Dec 31;116(4):541–62. doi: 10.1037/pspi0000154 (PMC6428189; doi:10.1037/pspi0000154)
Supplement: Supplementary file 1 [file SupplementalMaterialsSimilarities.zip › SupplementalMaterialsSimilarities.docx]

**A New Way to Look at the Data: Similarities Between Groups of People are Large and Important**

**Supplemental Materials -** **Table of Contents**

- **Materials for Study 1 (World Values Survey data)**
- **Alternative Measures of Similarity**
- **Tables of Detailed Results**
- **Figures Used in Study 5**
- **Additional Study: European Social Survey**
  - Method
  - Results
  - Tables of Detailed Results

# Materials for Study 1

Wordings of the items as reported in the official SPSS datasets of the World Values Survey (Study 1) and European Social Survey (Study 2). The exact wordings and positions in the questionnaire can be found on the webpages of the two surveys in question: (<http://www.worldvaluessurvey.org/WVSDocumentationWV6.jsp> and <http://www.europeansocialsurvey.org/data/download.html?r=6>)

## Measures in Study 1

Items used in WVS-factors

*Trust in people you know:* (1) How much you trust: Your family; (2) How much you trust: Your neighborhood; (3) How much you trust: People you know personally

*Trust in strangers*: (1) How much you trust: People you meet for the first time; (2) How much you trust: People of another religion; (3) How much you trust: People of another nationality.

*Understanding of democracy*: Democracy: Governments tax the rich and subsidize the poor; (2) Democracy: People choose their leaders in free elections; (3) Democracy: People receive state aid for unemployment; (4) Democracy: Civil rights protect people’s liberty from state oppression; (5) Democracy: The state makes people's incomes equal; (6) Democracy: Women have the same rights as men.

*Confidence in political institutions*: (1) [How much] Confidence [do you have in]: The police; (2) Confidence: The courts; (3) Confidence: The government (in your nation’s capital); (4) Confidence: Political Parties; (5) Confidence: Parliament; (6) Confidence: The civil service.

*Perceived respect of the own society towards elderly*: (1) People over 70: are seen as friendly [by own society]; (2) People over 70: are seen as competent [by own society]; (3) People over 70: viewed with respect [by own society].

*Ageism*: (1) Older people get more than their fair share from the government; (2) Older people are a burden on society; (3) Companies that employ young people perform better than those that employ people of different ages; (4) Old people have too much political influence.

*Trust in Science:* (1) Science and technology are making our lives healthier, easier, and more comfortable; (2) Because of science and technology, there will be more opportunities for the next generation; (3) The world is better off, or worse off, because of science and technology.

*Science skepticism*: (1) We depend too much on science and not enough on faith; (2) One of the bad effects of science is that it breaks down people’s ideas of right and wrong; (3) It is not important for me to know about science in my daily life.

*Attitudes towards personal-sexual issues*: (1) [Do you think it is] Justifiable: Homosexuality; (2) Justifiable: Prostitution; (3) Justifiable: Abortion; (4) Justifiable: Divorce; (5) Justifiable: Sex before marriage; (6) Justifiable: Suicide; (7) Justifiable: Euthanasia.

*Attitudes towards dishonest-illegal issues*: (1) Justifiable: Claiming government benefits to which you are not entitled; (2) Justifiable: Avoiding a fare on public transport; (3) Justifiable: Stealing property; (4) Justifiable: Cheating on taxes if you have a chance; (5) Justifiable: Someone accepting a bribe in the course of their duties.

*Attitudes towards (domestic) violence:* (1) Justifiable: For a man to beat his wife; (2) Justifiable: Parents beating children; (3) Justifiable: Violence against other people.

*Political attitudes*. Self-positioning on political scale from 1 (left) to 10 (right).

**Alternative Measures of Similarity**

## Percentage of common scores (PCS) – a non-parametric measure of similarity

The first of our two newly developed measures estimates similarity by calculating the *percentage of common scores* across two distributions. We label this the percentage of common scores (PCS) measure. The PCS can be used for both normal and non-normal distributions, and is easy to interpret. The PCS can be interpreted as the percentage of one group that has the same scale responses as the other group. For example, a percentage of 90 indicates that only 10 percent of each group has chosen a response on the measurement scale that is not mirrored in the other group.

PCS is computed using an easy to use function we developed in R (see below for the R code). In developing the method, two issues had to be addressed. First, the PCS for an item with a 5-point response scale is likely to be larger than for an item that has a 10-point response scale. To address this issue, we calculate the PCS by normalizing the data separately for each group, counting the number of values in the following five groups: 0 to 0.2, 0.2 to 0.4, 0.6 to 0.8, and 0.8 to 1. Each score in the first group would be replaced by 0.1, each score in the second group by 0.3, and so on. (The scores 0.1, 0.3, etc., are arbitrary numbers; alternatives such as 1 to 5 would have fulfilled the same purpose). Through this transformation, we ensure that each variable consists of different frequencies of the same five scores. That is, we standardized the number of scale points for all variables to 5. We applied this transformation to all measures, because many recent and reliable scales use at least a 5-point scale. A second issue is differences in sample size: If one sample is twice as large as the other, how can the PCS be calculated? To address this, we use bootstrapping methods. That is, from the larger group we randomly draw a sample of the same size as the smaller group and calculate the number of responses in common with the smaller group. After normalizing and clustering the values into one of the five groups as described above, we compute the percentage of common responses between the two vectors. This is done 100 times for each variable. The median of the 100 common responses is then used as an estimate of the PCS.

The interpretation of the PCS is approximately the same as the PCR when the two distributions are normal, especially if the sample sizes are equal. The primary difference is that the PCS refers directly to common scale responses across two groups. That is, a research report could indicate that one group was X units higher than the other, but gave the same responses Y% of the time (rather than report a difference of X and overlap of Y). Furthermore, the PCS returns, on average, a more conservative estimate of similarity (cf. results in the main manuscript). Given that PCR and PCS are conceptually similar, we expected large correlations between these measures. Indeed, all correlations were as expected, both on a single pairwise comparison level and on an aggregate level. For example, within the category of country, PCR and PCS of moral attitudes towards personal-sexual behaviors correlated very strongly, *r*[1768] = .98. On an aggregate level (correlating the medians of each variable across all categories), PCR was slightly but reliably larger (*M* = 93.30, *median =* 95.00, *SD* = 5.37) than PCS (*M* = 91.23, *median* = 93.50, *SD* = 6.46), on average, *t*(131) = 11.96, *p* < .001, but the medians of PCR and PCS correlated highly, r(130) = .96.

There are other effect sizes that could be used to estimate similarity instead of the ones we have used. We consider all as interesting alternatives. One option is the intra-class correlation (ICC[1]; Bliese, 2000). The ICC(1) uses the between-group and within-group variance directly. It can be interpreted as the average proportion of total variance explained by group membership within categories, such as countries or age groups. An ICC(1) of .2 indicates, for example, that 20 percent of a variable can be explained by group membership, or, alternatively, that 80 percent of the variation cannot be explained by group membership. If similarities are large, between group variance is low and the ICC(1) is therefore close to zero. However, the concept of amount of explained variance is not very intuitive and does not express similarities directly. Further, because the ICC(1) is calculated from an ANOVA model, the ICC(1) is a parametric measure.

A second option is the probability of superiority (PS) which is a non-parametric generalization of the common language effect size (McGraw & Wong, 1992). The PS returns the probability that a randomly selected person from the higher-scoring group has a higher value than a randomly selected person from the other group (Ruscio, 2008; Ruscio & Mullen, 2012). A PS of .50, for example, would indicate maximum similarity, whereas values close to 0 or 1 would indicate large differences. We think that PS is an underused measure to present differences. However, the “probability of superiority” is not a label that could be applied to directly convey similarities.

A third alternative is to smooth a kernel density estimate over the data and compute the overlap of the two functions. Figure 1B displays such a possibility. This approach can be understood as a non-parametric version of the overlapping coefficient (Schmid & Schmidt, 2006). However, an evident problem is that the distributions of both the data and the response options vary. Hence, smoothing a kernel density estimation over all data in the same way becomes practically impossible. For example, assume the data in both groups are normally distributed. When the kernel density estimates are smoothed in a loose way above the data, we obtain two normal distributions. If we were to smooth the density exactly above the data, the result would be the same as for the PCS measure, without standardizing it to five response options.

As a fourth alternative, Lalonde et al. (2015) have suggested using equivalence-based procedures as “valid tests of similarity between groups” (p. 529). Specifically, they tested whether the standardized group differences can be considered to be equivalent (e.g., Cribbie, Gruman, & Arpin-Cribbie, 2004; Lakens, 2017). However, equivalence tests test whether group means are equivalent, not whether groups are similar or equivalent. That is, mean differences can be non-equivalent, but still similar, because equivalence is often rejected when the confidence intervals of, for example, Cohen’s *d* cross a threshold of 0.30, 0.50, or, rather rarely, 0.80 (Lakens, 2017). As demonstrated in the main manuscript, all three thresholds still suggest substantial similarities (PCR ≥ 69). Thus, equivalence tests are only suitable to test whether means are similar or equivalent, but not groups.

A somewhat distinct type of analysis which also focuses on similarities (also referred to as congruencies or matching) is response surface analysis (RSA), which is based on polynomial regression (Barranti, Carlson, & Côté, 2017; Edwards, 2002; Edwards & Cable, 2009). However, RSA relies on a different analytical approach and is very distinct from our approach to describing similarities, and indeed all the other approaches described above, because “RSA assesses whether (mis)matches matter by modeling how all possible combinations of two predictors are associated with an outcome and does so in three-dimensional space” (Barranti et al., 2017, p. 1f). In other words, RSA needs one variable more – the outcome variable – than our approach and addresses very different research questions, including “Do similar individuals like each other more or less than do dissimilar individuals?” or “Is well-being related to a match between personal and cultural values?” (Barranti et al., 2017, p. 2).

For all of these approaches, we submit that the interpretation is less direct and intuitive than an approach that simply ranges from 0 to 100 percent, which can be easily obtained through the PCR (i.e., a non-linear transformation of Cohen’s *d*). Moreover, none of the existing alternatives takes the absolute scale range into account, which is intrinsic to the AE estimate.

**Tables of Detailed Results for Study 1**

Table S1

*Comparisons of the 10 value types and 12 other variables between countries*

|  | Cohen’s *d* | | PCR | | | | AE | |
| --- | --- | --- | --- | --- | --- | --- | --- | --- |
|  | Med *d* | Max. *d* | Med | ≥ .90 | ≥ .95 | Min | Med AE | Max AE |
| Security | .37 | 1.76 | 85 | 36 | 19 | 38 | 20 | 40 |
| Tradition (25) | .41 | 2.65 | 84 | 31 | 16 | 19 | 20 | 60 |
| Conformity (4) | .32 | 2.03 | 87 | 40 | 20 | 31 | 20 | 60 |
| Benevolence (2) | .35 | 2.42 | 86 | 35 | 18 | 23 | 20 | 60 |
| Universalism | .32 | 1.87 | 87 | 40 | 21 | 35 | 20 | 40 |
| Self-direction (1) | .29 | 1.74 | 88 | 43 | 23 | 38 | 20 | 60 |
| Stimulation (8) | .32 | 2.05 | 87 | 39 | 20 | 31 | 20 | 60 |
| Hedonism (32) | .45 | 1.98 | 82 | 29 | 15 | 32 | 20 | 60 |
| Achievement (33) | .41 | 2.63 | 84 | 31 | 15 | 19 | 20 | 80 |
| Power (46) | .50 | 2.40 | 80 | 26 | 13 | 23 | 20 | 60 |
| Trust in known people | .34 | 1.83 | 86 | 37 | 19 | 36 | 0 | 33 |
| Trust in strangers (2) | .41 | 2.22 | 84 | 34 | 17 | 27 | 11 | 56 |
| Understanding of democracy | .39 | 1.98 | 84 | 35 | 18 | 32 | 09 | 39 |
| Confidence in political institutions (46) | .51 | 3.09 | 80 | 26 | 12 | 12 | 11 | 64 |
| Perceived respect for elderly | .31 | 2.19 | 88 | 41 | 23 | 27 | 08 | 50 |
| Ageism | .47 | 1.85 | 82 | 27 | 15 | 36 | 08 | 33 |
| Trust in science | .31 | 1.60 | 88 | 40 | 23 | 42 | 07 | 37 |
| Skepticism towards science | .36 | 1.81 | 86 | 37 | 19 | 36 | 07 | 37 |
| Morality: attitudes towards personal-sexual issues (75) | .73 | 3.79 | 71 | 19 | 9 | 6 | 17 | 67 |
| Morality: attitudes towards dishonest-illegal issues | .36 | 1.87 | 86 | 36 | 18 | 35 | 07 | 33 |
| Morality: attitudes towards (domestic) violence | .44 | 3.42 | 83 | 32 | 17 | 9 | 07 | 41 |
| Left-right attitude | .25 | 1.43 | 90 | 50 | 30 | 47 | 11 | 33 |
| Average | .39 | 2.21 | 84 | 35 | 18 | 29 | 14 | 50 |

*Note*. PCR: Percentage of common responses, AE: absolute effect in percent. Med: Median, ≥ 90/95: Percentage of pairs with a PCR ≥ 90/95 percent. Numbers in brackets indicate the number of countries that differ significantly (absolute difference).

Table S2

*Comparisons of the 10 value types and 12 other variables between seven religious denominations*

|  | Cohen’s *d* | | PCR | | | | AE | |
| --- | --- | --- | --- | --- | --- | --- | --- | --- |
|  | Med *d* | Max. *d* | Med | ≥ .90 | ≥ .95 | Min | Med AE | Max AE |
| Security | .19 | .43 | 93 | 67 | 38 | 83 | 0 | 0 |
| Tradition | .20 | .67 | 92 | 52 | 19 | 74 | 0 | 20 |
| Conformity | .19 | .60 | 93 | 62 | 24 | 77 | 0 | 20 |
| Benevolence | .12 | .35 | 95 | 76 | 52 | 86 | 0 | 20 |
| Universalism | .12 | .35 | 95 | 76 | 52 | 86 | 0 | 20 |
| Self-direction | .15 | .49 | 94 | 71 | 38 | 80 | 0 | 20 |
| Stimulation | .27 | .68 | 89 | 48 | 19 | 73 | 20 | 40 |
| Hedonism | .22 | .52 | 91 | 57 | 24 | 80 | 0 | 20 |
| Achievement | .20 | .59 | 92 | 62 | 29 | 77 | 20 | 20 |
| Power | .34 | .74 | 87 | 33 | 24 | 71 | 20 | 40 |
| Trust in known people | .17 | .44 | 93 | 71 | 33 | 83 | 0 | 11 |
| Trust in strangers | .21 | .59 | 92 | 62 | 29 | 77 | 0 | 11 |
| Understanding of democracy | .29 | .88 | 88 | 43 | 24 | 66 | 7 | 20 |
| Confidence in political institutions | .28 | .66 | 89 | 43 | 24 | 74 | 6 | 17 |
| Perceived respect for elderly | .14 | .29 | 94 | 90 | 43 | 89 | 8 | 8 |
| Ageism | .49 | 1.02 | 84 | 29 | 14 | 61 | 8 | 17 |
| Trust in science | .27 | .61 | 89 | 43 | 24 | 76 | 7 | 15 |
| Skepticism towards science | .11 | .27 | 96 | 95 | 57 | 89 | 4 | 7 |
| Morality: attitudes towards personal-sexual issues | .31 | 1.06 | 88 | 43 | 24 | 60 | 8 | 23 |
| Morality: attitudes towards dishonest-illegal issues | .29 | .82 | 95 | 71 | 48 | 68 | 4 | 20 |
| Morality: attitudes towards (domestic) violence | .29 | 1.17 | 88 | 38 | 19 | 56 | 7 | 22 |
| Left-right attitude | .14 | .35 | 94 | 86 | 48 | 86 | 0 | 11 |
| Average | .23 | .62 | 91 | 59 | 32 | 76 | 5 | 18 |

*Note*. PCR: Percentage of common responses, AE: absolute effect in percent. Med: Median, ≥ 90/95: Percentage of pairs with a PCR ≥ 90/95 percent.

Table S3

*Comparisons of the 10 value types and 12 other variables between different income classes*

|  | Cohen’s *d* | | PCR | | | | AE | |
| --- | --- | --- | --- | --- | --- | --- | --- | --- |
|  | Med *d* | Max. *d* | Med | ≥ .90 | ≥ .95 | Min | Med AE | Max AE |
| Security | .03 | .11 | 99 | 100 | 100 | 96 | 0 | 0 |
| Tradition | .05 | .18 | 98 | 100 | 91 | 93 | 0 | 0 |
| Conformity | .04 | .12 | 99 | 100 | 100 | 95 | 0 | 0 |
| Benevolence | .08 | .27 | 97 | 98 | 73 | 89 | 0 | 0 |
| Universalism | .05 | .17 | 98 | 100 | 76 | 93 | 0 | 0 |
| Self-direction | .15 | .37 | 94 | 69 | 38 | 85 | 20 | 20 |
| Stimulation | .18 | .41 | 93 | 67 | 38 | 84 | 20 | 20 |
| Hedonism | .15 | .36 | 94 | 73 | 40 | 86 | 0 | 0 |
| Achievement | .14 | .35 | 94 | 71 | 38 | 86 | 0 | 20 |
| Power | .20 | .50 | 92 | 58 | 27 | 80 | 20 | 40 |
| Trust in known people | .08 | .25 | 97 | 100 | 69 | 90 | 0 | 0 |
| Trust in strangers | .13 | .38 | 95 | 87 | 49 | 85 | 0 | 11 |
| Understanding of democracy | .11 | .27 | 96 | 98 | 60 | 89 | 2 | 6 |
| Confidence in political institutions | .19 | .47 | 93 | 64 | 33 | 81 | 6 | 14 |
| Perceived respect for elderly | .08 | .22 | 97 | 100 | 78 | 91 | 0 | 8 |
| Ageism | .09 | .29 | 97 | 87 | 58 | 88 | 0 | 0 |
| Trust in science | .19 | .46 | 92 | 67 | 36 | 82 | 4 | 11 |
| Skepticism towards science | .06 | .15 | 97 | 100 | 84 | 94 | 4 | 4 |
| Morality: attitudes towards personal-sexual issues | .06 | .27 | 98 | 98 | 78 | 89 | 2 | 6 |
| Morality: attitudes towards dishonest-illegal issues | .09 | .33 | 96 | 91 | 64 | 87 | 2 | 4 |
| Morality: attitudes towards (domestic) violence | .09 | .18 | 97 | 100 | 69 | 93 | 4 | 8 |
| Left-right attitude | .17 | .55 | 93 | 67 | 33 | 78 | 11 | .22 |
| Average | .11 | .30 | 96 | 86 | 61 | 88 | 4 | 9 |

*Note*. PCR: Percentage of common responses, AE: absolute effect in percent. Med: Median, ≥ 90/95: Percentage of pairs with a PCR ≥ 90/95 percent.

Table S4

*Comparisons of the 10 value types and 12 other variables between educational groups*

|  | Cohen’s *d* | | PCR | | | | AE | |
| --- | --- | --- | --- | --- | --- | --- | --- | --- |
|  | Med *d* | Max. *d* | Med | ≥ .90 | ≥ .95 | Min | Med AE | Max AE |
| Security | .06 | .21 | 98 | 100 | 83 | 92 | 0 | 0 |
| Tradition | .15 | .39 | 94 | 81 | 42 | 84 | 0 | 0 |
| Conformity | .06 | .17 | 98 | 100 | 94 | 93 | 0 | 0 |
| Benevolence | .07 | .21 | 97 | 100 | 86 | 92 | 0 | 0 |
| Universalism | .05 | .14 | 98 | 100 | 94 | 94 | 0 | 0 |
| Self-direction | .13 | .40 | 95 | 92 | 50 | 84 | 10 | 20 |
| Stimulation | .13 | .28 | 95 | 92 | 47 | 89 | 0 | 20 |
| Hedonism | .07 | .29 | 97 | 97 | 69 | 88 | 0 | 0 |
| Achievement | .04 | .11 | 98 | 100 | 100 | 96 | 0 | 0 |
| Power | .06 | .16 | 97 | 100 | 86 | 94 | 0 | 0 |
| Trust in known people | .07 | .24 | 97 | 100 | 67 | 90 | 0 | 0 |
| Trust in strangers | .15 | .42 | 94 | 81 | 42 | 83 | 0 | 11 |
| Understanding of democracy | .07 | .21 | 97 | 100 | 81 | 92 | 2 | 4 |
| Confidence in political institutions | .04 | .12 | 98 | 100 | 100 | 95 | 6 | 6 |
| Perceived respect for elderly | .04 | .15 | 98 | 100 | 97 | 94 | 0 | 8 |
| Ageism | .13 | .48 | 95 | 83 | 47 | 81 | 0 | 11 |
| Trust in science | .14 | .39 | 95 | 89 | 47 | 85 | 4 | 7 |
| Skepticism towards science | .12 | .36 | 95 | 86 | 56 | 86 | 4 | 7 |
| Morality: attitudes towards personal-sexual issues | .23 | .67 | 91 | 52 | 25 | 74 | 6 | 19 |
| Morality: attitudes towards dishonest-illegal issues | .08 | .21 | 97 | 100 | 86 | 92 | 2 | 3 |
| Morality: attitudes towards (domestic) violence | .12 | .46 | 95 | 83 | 61 | 82 | 4 | 11 |
| Left-right attitude | .05 | .18 | 98 | 100 | 89 | 93 | 0 | 11 |
| Average | .09 | .28 | 96 | 93 | 70 | 89 | 2 | 6 |

*Note*. PCR: Percentage of common responses, AE: absolute effect in percent. Med: Median, ≥ 90/95: Percentage of pairs with a PCR ≥ 90/95 percent.

Table S5

*Comparisons of the 10 value types and 12 other variables between gender*

|  | Cohen’s *d* | | PCR | | | | AE | |
| --- | --- | --- | --- | --- | --- | --- | --- | --- |
|  | Med *d* | Max. *d* | Med | ≥ .90 | ≥ .95 | Min | Med AE | Max AE |
| Security | .08 |  | 97 |  |  |  | 0 |  |
| Tradition | .06 |  | 98 |  |  |  | 0 |  |
| Conformity | .04 |  | 98 |  |  |  | 0 |  |
| Benevolence | .01 |  | 100 |  |  |  | 0 |  |
| Universalism | .04 |  | 98 |  |  |  | 0 |  |
| Self-direction | .11 |  | 95 |  |  |  | 20 |  |
| Stimulation | .25 |  | 90 |  |  |  | 20 |  |
| Hedonism | .08 |  | 97 |  |  |  | 0 |  |
| Achievement | .14 |  | 95 |  |  |  | 0 |  |
| Power | .14 |  | 94 |  |  |  | 0 |  |
| Trust in known people | .04 |  | 98 |  |  |  | 0 |  |
| Trust in strangers | .04 |  | 98 |  |  |  | 0 |  |
| Understanding of democracy | .07 |  | 97 |  |  |  | 2 |  |
| Confidence in political institutions | .03 |  | 99 |  |  |  | 2 |  |
| Perceived respect for elderly | .01 |  | 100 |  |  |  | 0 |  |
| Ageism | .10 |  | 96 |  |  |  | 0 |  |
| Trust in science | .05 |  | 98 |  |  |  | 4 |  |
| Skepticism towards science | .06 |  | 98 |  |  |  | 4 |  |
| Morality: attitudes towards personal-sexual issues | .00 |  | 100 |  |  |  | 0 |  |
| Morality: attitudes towards dishonest-illegal issues | .06 | . | 98 |  |  |  | 2 |  |
| Morality: attitudes towards (domestic) violence | .12 |  | 95 |  |  |  | 4 |  |
| Left-right attitude | .01 |  | 99 |  |  |  | 0 |  |
| Average | .07 |  | 97 |  |  |  | 3 |  |

*Note*. PCR: Percentage of common responses, AE: absolute effect in percent. Med: Median, ≥ 90/95: Percentage of pairs with a PCR ≥ 90/95 percent.

Table S6

*Comparisons of the 10 value types and 12 other variables between age cohorts*

|  | Cohen’s d | | PCR | | | | AE | |
| --- | --- | --- | --- | --- | --- | --- | --- | --- |
|  | Med *d* | Max. *d* | Med | ≥ .90 | ≥ .95 | Min | Med AE | Max AE |
| Security | .05 | .12 | 98 | 100 | 100 | 95 | 0 | 0 |
| Tradition | .05 | .13 | 98 | 100 | 98 | 95 | 0 | 0 |
| Conformity | .03 | .10 | 99 | 100 | 100 | 96 | 0 | 0 |
| Benevolence | .05 | .17 | 98 | 100 | 93 | 93 | 0 | 0 |
| Universalism | .03 | .07 | 99 | 100 | 100 | 97 | 0 | 0 |
| Self-direction | .18 | .52 | 93 | 71 | 36 | 80 | 20 | 20 |
| Stimulation | .30 | .90 | 88 | 42 | 18 | 65 | 20 | 40 |
| Hedonism | .21 | .73 | 91 | 56 | 29 | 71 | 20 | 40 |
| Achievement | .25 | .75 | 90 | 49 | 22 | 71 | 0 | 20 |
| Power | .26 | .75 | 90 | 49 | 20 | 71 | 20 | 40 |
| Trust in known people | .09 | .30 | 97 | 93 | 69 | 88 | 0 | 0 |
| Trust in strangers | .07 | .23 | 97 | 100 | 78 | 91 | 0 | 0 |
| Understanding of democracy | .08 | .25 | 97 | 100 | 73 | 90 | 2 | 6 |
| Confidence in political institutions | .03 | .12 | 99 | 100 | 100 | 95 | 0 | 6 |
| Perceived respect for elderly | .03 | .08 | 99 | 100 | 100 | 97 | 0 | 0 |
| Ageism | .15 | .49 | 94 | 71 | 42 | 81 | 0 | 8 |
| Trust in science | .04 | .15 | 98 | 100 | 93 | 94 | 0 | 4 |
| Skepticism towards science | .02 | .09 | 99 | 100 | 100 | 96 | 0 | 4 |
| Morality: attitudes towards personal-sexual issues | .05 | .15 | 98 | 100 | 98 | 94 | 2 | 4 |
| Morality: attitudes towards dishonest-illegal issues | .14 | .46 | 94 | 76 | 40 | 82 | 2 | 9 |
| Morality: attitudes towards (domestic) violence | .16 | .46 | 94 | 76 | 40 | 82 | 4 | .11 |
| Left-right attitude | .03 | .07 | 99 | 100 | 100 | 97 | 0 | .11 |
| Average | .10 | .32 | 96 | 86 | 70 | 87 | 4 | .10 |

*Note*. PCR: Percentage of common responses, AE: absolute effect in percent. Med: Median, ≥ 90/95: Percentage of pairs with a PCR ≥ 90/95 percent.

# Figures Used in Study 5

The following Figures were used in Study 5.

## Figures displaying the Moral Foundations
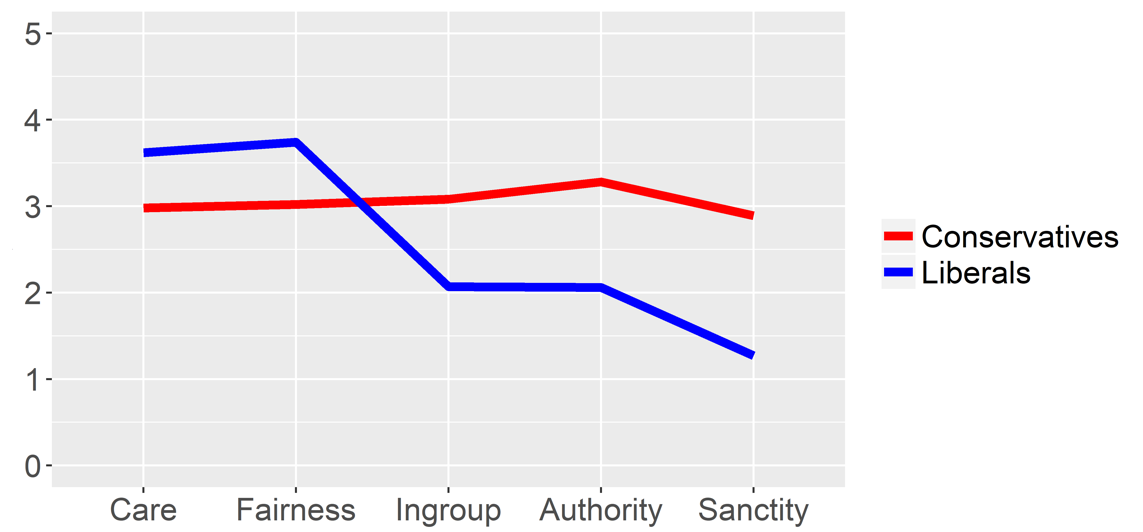


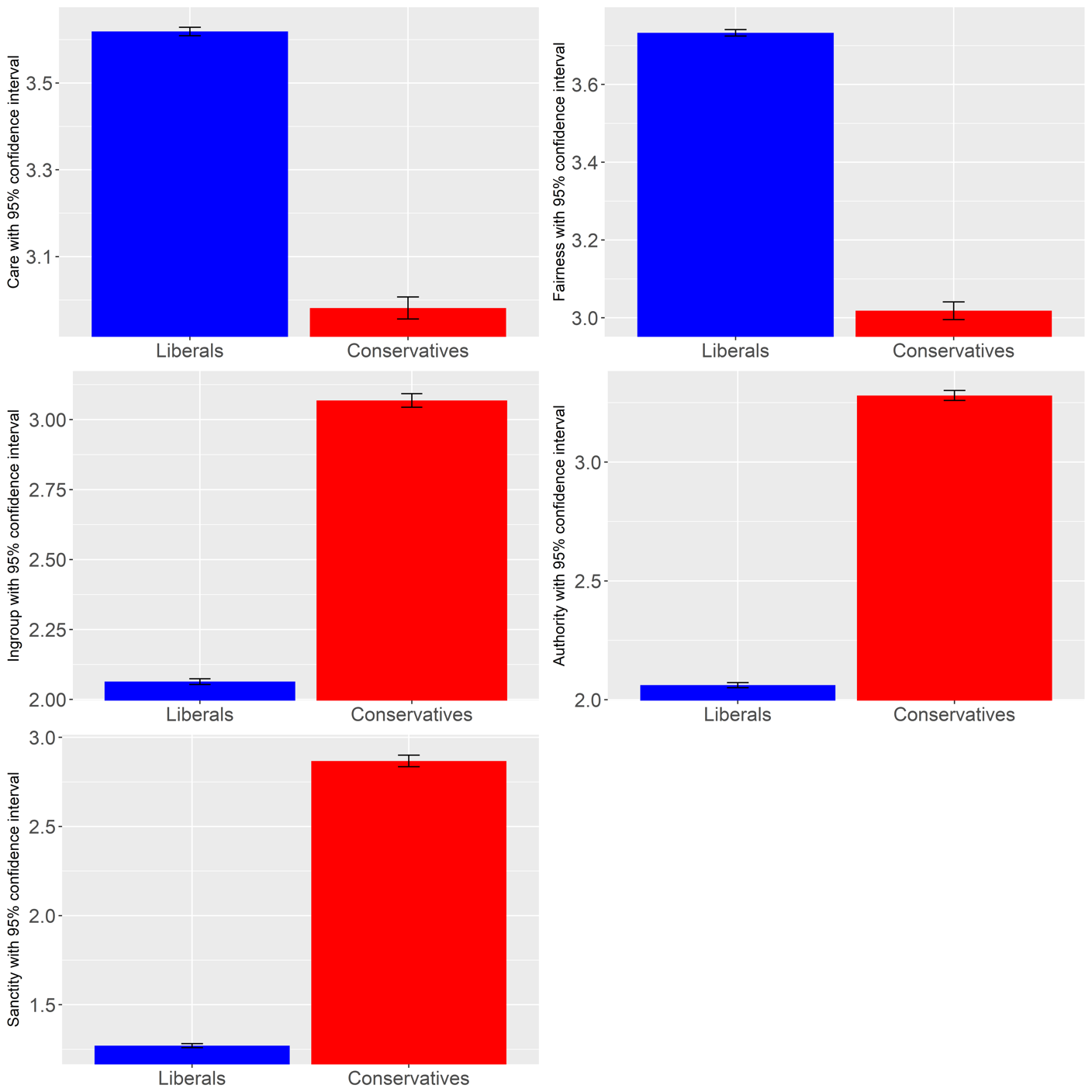

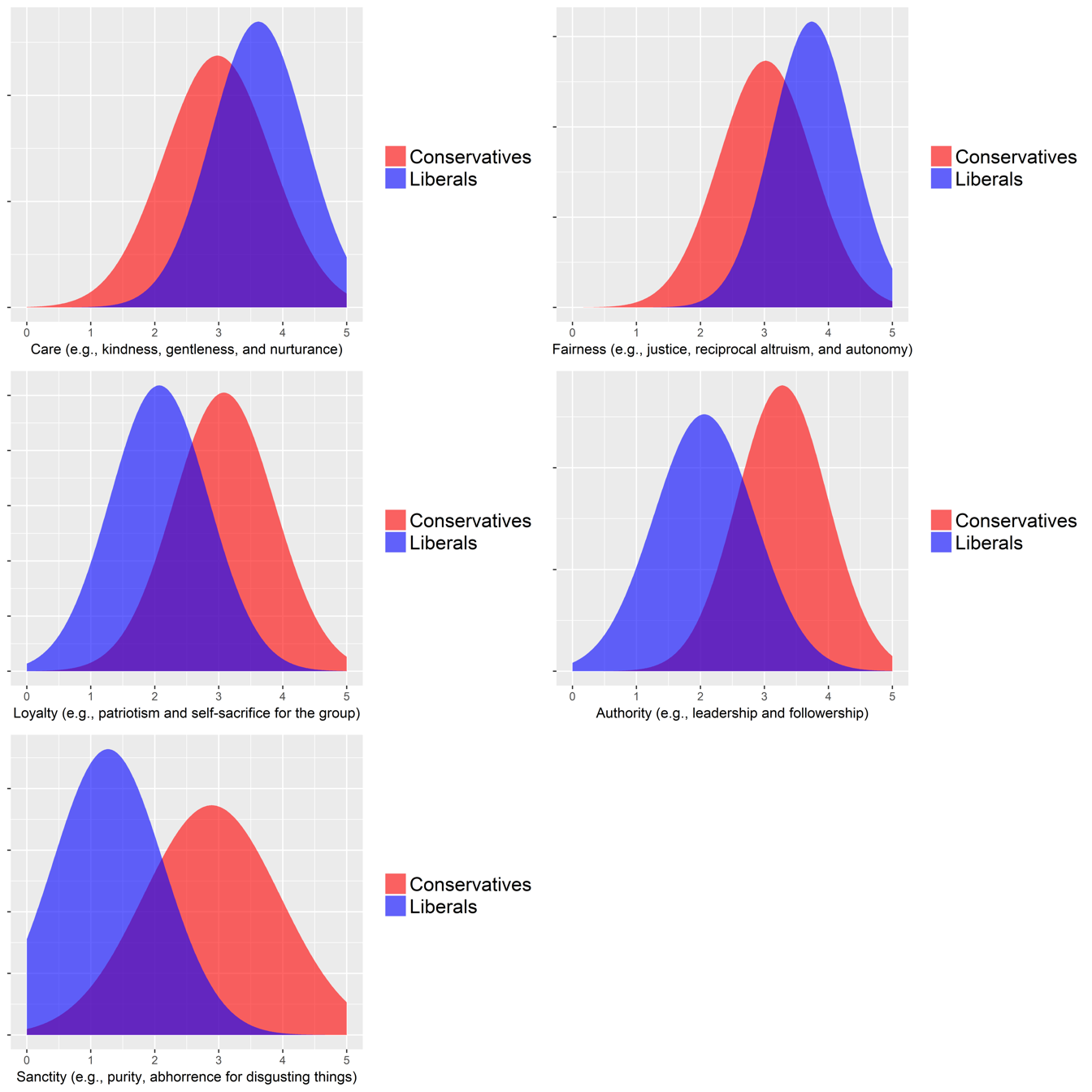


## Figures Displaying the IQ of Religious Believers and non-Believers
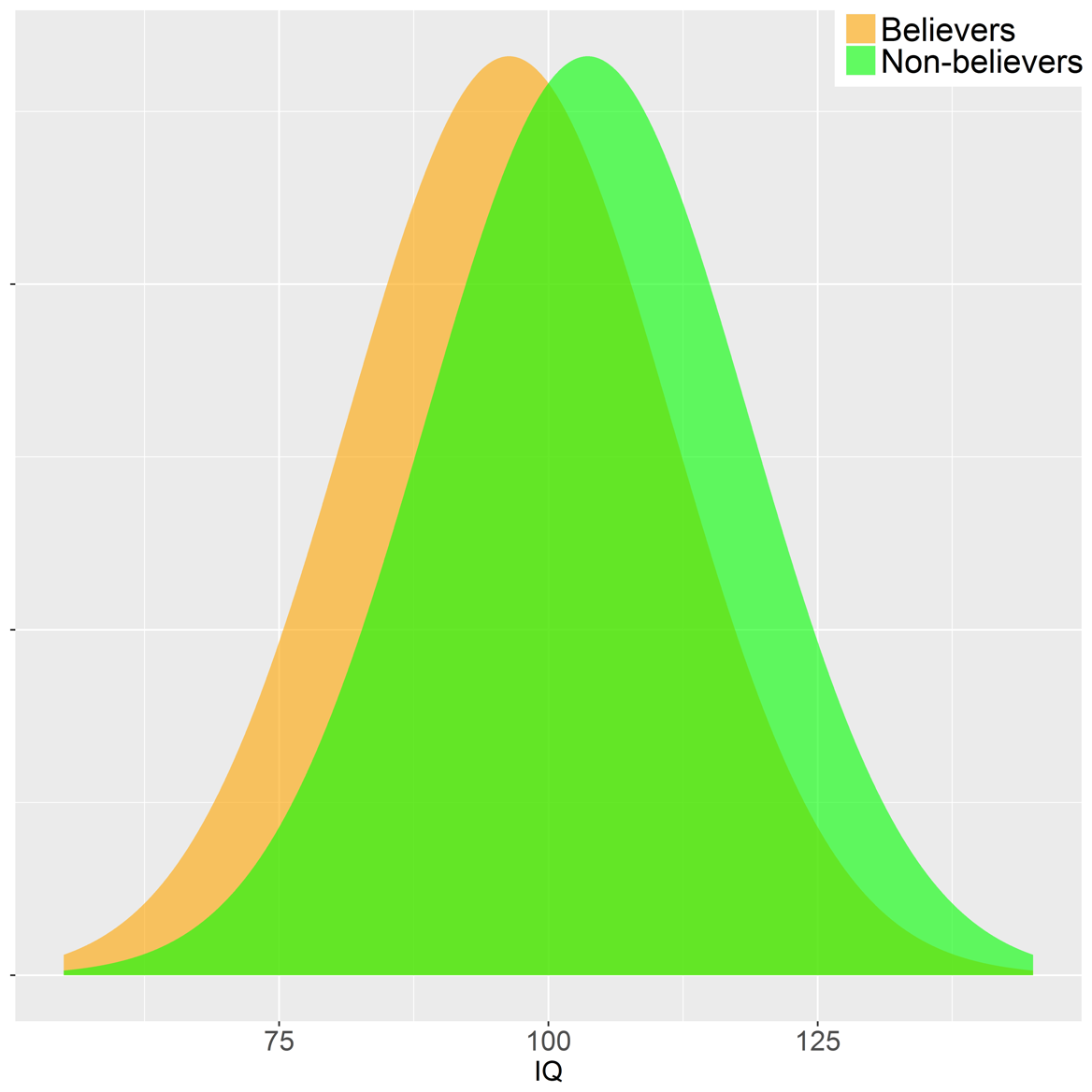


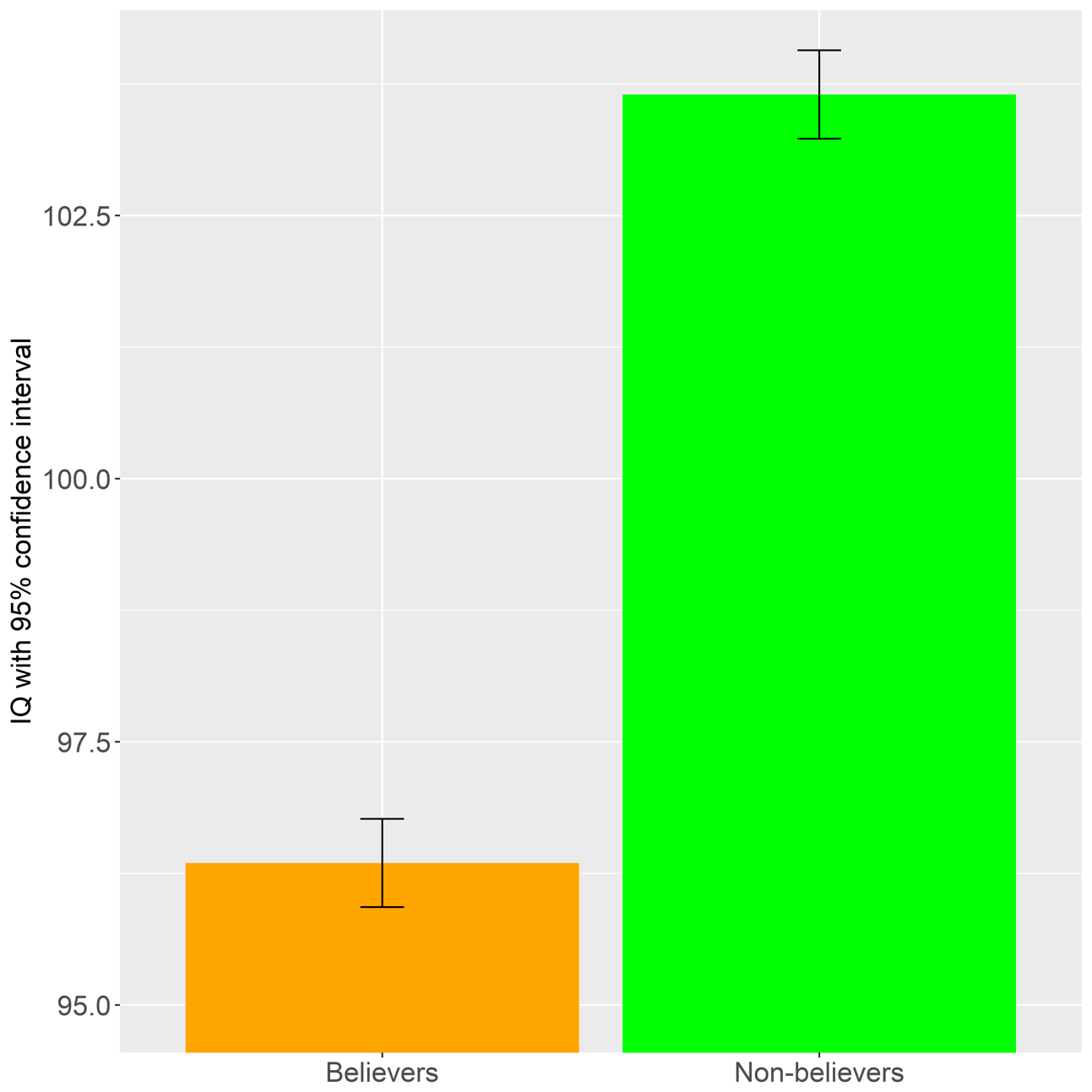


# Additional Study: European Social Survey

In the additional study, we used the data provided by the European Social Survey (ESS; www.europeansocialsurvey.org), 6^th^ round, collected in 2012 and 2013. The ESS has been conducted every other year since 2002 in European and adjacent (Turkey, Israel) countries. The number of countries included ranges from 20 to 31. The survey contains a variety of items that the survey designers selected *a priori* based on the items’ perceived relevance for understanding differences between countries. For the sake of conceptual parsimony in our analyses, we categorized these items into several factors, as described below.

## Method

*Participants*. The sample contained 54,673 participants who were representative of 29 different countries. Prior to the analyses, 591 participants were excluded from all of the analyses because they did not respond to four or more items on the Portrait Value Questionnaire (PVQ-21; Schwartz et al., 2001), leaving 54,082 participants. The mean age of the sample was 48.23 years (*SD* = 18.56, *range* = 15-103), including 29,395 women (54.35%).

*Material and procedure*. The five categories we examined were country, gender, income, education, and age. For country, we used all 29 countries in the ESS. Gender was measured dichotomously as male or female. Income was measured in a country-specific way, by coding the total household’s net income into the respective decile of the income distribution for the household’s nation. To measure education, the International Standard Classification of Education (ISCED) was used, splitting the participants into seven groups, ranging from less than lower secondary (*n* = 5753) to higher tertiary education (*n* = 6674). The continuous variable ‘year of birth’ was split into 10 equal sized groups, ranging from born in 1939 or before to born in 1991 or later.

In total, 24 dependent variables were chosen. We used all 21 items of the Portrait Value Questionnaire (PVQ), which measures the 10 value types according to Schwartz’s (Schwartz, 1992) model of human values (Schwartz, 2003; Schwartz et al., 2001): security, tradition, conformity, benevolence, universalism, self-direction, stimulation, hedonism, achievement, and power (Schwartz, 1992). Using a scale from 1 (very much like me) to 6 (not like me at all), participants indicated how similar they were to a fictitious person who shows a positive attitude towards a prototypical behavior for one of the ten value types. Examples for items include “Thinking up new ideas and being creative is important to her/him. She/he likes to do things in her/his own original way” (self-direction) and “It is important to her/him to be rich. She/he wants to have a lot of money and expensive things” (power).

The remaining 151 items in the ESS that were measured on a quasi-interval level were factor analyzed with a principal component analysis (PCA). After varimax rotation, we obtained 13 factors, consisting of 67 items. Many of these items have been used in international comparisons and in studies examining the ESS in particular (e.g., Davidov & Meuleman, 2012; Grönlund & Setala, 2012; Marozzi, 2014; Visser, Scholte, & Scheepers, 2013). We labeled these factors as engagement in different political activities (6 items, α = .64), attitudes towards immigrants (6, α = .89), pessimistic world view (4, α = .61), religiosity (4, α = .85), feeling depressed (7, α = .84), subjective happiness (4, α = .79), trust in other people (3, α = .78), trust in political institutions (7, α = .91), feeling optimistic and fulfilled (6, α = .76), relationship to neighbors (3, α = .66), relationship to other people you are close to (3, α = .72), trust in democratic rights (7, α = .85), and trust in democratic processes (6, α = .83). Items had to load at least .50 on one component and less than .40 on any other component (with a loading difference between the two highest loading components of at least .25) to be included in a component.

## Details of Measures

To describe the computation of our factors, we report below the SPSS labels as provided in the data file of the ESS.

*Political activities.* (1) Contacted politician or government official last 12 months; (2) Worked in political party or action group last 12 months; (3) Worked in another organisation or association last 12 months; (4) Worn or displayed campaign badge/sticker last 12 months; (5) Signed petition last 12 months; (6) Taken part in lawful public demonstration last 12 months.

*Attitudes towards immigrants.* (1) Allow many/few immigrants of same race/ethnic group as majority; (2) Allow many/few immigrants of different race/ethnic group from majority; (3) Allow many/few immigrants from poorer countries outside Europe; (4) Immigration bad or good for country's economy; (5) Country's cultural life undermined or enriched by immigrants; (6) Immigrants make country worse or better place to live.

*Feeling depressed*. (1) Felt depressed, how often past week; (2) Felt everything did as effort, how often past week; (3) Sleep was restless, how often past week; (4) Felt lonely, how often past week; (5) Felt sad, how often past week; (6) Could not get going, how often past week; (7) Felt anxious, how often past week.

*Subjective happiness*. (1) Were happy, how often past week; (2) Enjoyed life, how often past week; (3) Had lot of energy, how often past week; (4) Felt calm and peaceful, how often past week.

*Feeling optimistic*. (1) Always optimistic about my future; (2) In general feel very positive about myself; (3) Free to decide how to live my life; (4) Feel accomplishment from what I do; (5) There are a lot of things I am good at; (6) Feel what I do in life is valuable and worthwhile.

*Pessimistic worldview*. (1) Little chance to show how capable I am; (2) When things go wrong in my life it takes a long time to get back to normal; (3) Hard to be hopeful about the future of the world; (4) For most people in country life is getting worse.

*Trust in other people* (bipolar scale). (1) Most people can be trusted vs you can't be too careful; (2) Most people try to take advantage of you vs try to be fair; (3) Most of the time people helpful vs mostly looking out for themselves.

*Trust in political institutions*. (1) Trust in the country's parliament; (2) Trust in the legal system; (3) Trust in the police; (4) Trust in politicians; (5) Trust in political parties; (6) Trust in the European Parliament; (7) Trust in the United Nations.

*Relationships with neighbors.* (1) Feel people in local area help one another; (2) Feel people treat you with respect; (3) Feel close to the people in local area.

*Relationships with other people*. (1) Feel appreciated by people you are close to; (2) Receive help and support from people you are close to; (3) Provide help and support to people you are close to.

*Trust in democratic rights*. (1) Citizens have the final say on political issues by voting directly in referendums; (2) The courts treat everyone the same; (3) The courts are able to stop the government acting beyond its authority; (4) Governing parties are punished in elections when they have done a bad job; (5) The government protects all citizens against poverty; (6) The government explains its decisions to voters; (7) The government takes measures to reduce differences in income levels.

*Trust in democratic processes*. (1) National elections are free and fair; (2) Voters discuss politics with people they know before deciding how to vote; (3) Different political parties offer clear alternatives to one another; (4) Opposition parties are free to criticise the government; (5) The media are free to criticise the government; (6) The media provide citizens with reliable information to judge the government.

*Political attitudes*. Placement on a political left right scale from 0 (left) to 10 (right).

*Religiosity*. (1) Belonging to a particular religion or denomination; (2) How religious are you? (3) How often do you attend religious services apart from special occasions? (4) How often do you pray apart from religious services?

## Results

First, we report validations of the four statistics measuring similarity. Next, important findings for each of the five categories are reported. We then examine which categories reveal larger similarities across all of the dependent variables and which of the dependent variables show larger similarities across all categories.

**Validation of the measures used**. First, we validated the measures across all categories and variables by correlating them with each other to estimate their convergent and divergent validity. Given that PCR and PCS are conceptually more similar to each other than to the other two measures, we expected larger correlations between PCR and PCS than with the AE. Indeed, the medians of PCR and PCS across all categories variables correlated highly (*r*[118] = .95), but, as expected, PCR was slightly but reliably larger (*M* = 93.01, *SD* = 4.75) than PCS *(M* = 92.08, *SD* = 5.00) on average, *t*(119) = 6.47, *p* < .001, replicating the findings of Study 1.

**Overall findings within each category**. As expected, the median values for the five statistics revealed high levels of similarity. Only 17 out of the 29,464 pairwise comparisons revealed a PCR of below 50 percent and an AE of above 50%. For all variables, the smallest PCR between two groups is well above 50 percent, and the AE below 30%. Thus, even between extreme groups (e.g., highly vs lower educated), large similarities were found. With a few exceptions (see examples below), people across the different groups within each category revealed on average the same amount of similarity for the 10 value types as for the 14 other variables.

***Countries***. The median for all 24 dependent variables indicates large similarities between countries. The average PCR was 87. That is, on average, 87 percent of the participants in two countries chose an answer that was mirrored in the other country. The average absolute effect was .08, indicating that median differences between two countries were less than one tenth of the response scale. For example, the median PCR was 84 for the security value type. The median AE for this value was 10%, and the largest AE between two countries was 40% (see Table S7).

There was some variation in the amount of similarity between the variables. For religiosity, for example, the amount of similarity was of moderate size (median PCR = 79, median AE = 19%). Also, for 17 pairwise comparisons the differences were larger than the similarities. For example, the largest differences were found between Czech Republic as the least religious country (*M* = 1.67, median = 0.42), on the one hand, and Poland and Cyprus, as the least religious countries, on the other hand (*M*s ≥ 6.74, both medians = 7.58), resulting in PCRs of around 22 and AEs of 72%.

Table S7

*Comparisons of the 10 value types and 14 other variables between countries*

|  | Cohen’s *d* | | PCR | | | | AE | |
| --- | --- | --- | --- | --- | --- | --- | --- | --- |
|  | Med *d* | Max. *d* | Med | ≥ .90 | ≥ .95 | Min | Med AE | Max AE |
| Security | .41 | 1.62 | 84 | 32 | 16 | 42 | 10 | 40 |
| Tradition | .34 | 1.27 | 86 | 38 | 19 | 53 | 10 | 20 |
| Conformity | .28 | 1.28 | 89 | 46 | 23 | 52 | 10 | 30 |
| Benevolence | .31 | 1.50 | 88 | 42 | 21 | 45 | 10 | 30 |
| Universalism | .30 | 1.37 | 88 | 44 | 23 | 49 | 7 | 27 |
| Self-direction | .24 | .95 | 90 | 50 | 25 | 64 | 10 | 20 |
| Stimulation | .15 | .68 | 94 | 74 | 42 | 73 | 10 | 20 |
| Hedonism | .33 | .98 | 87 | 40 | 19 | 62 | 10 | 30 |
| Achievement | .40 | 1.64 | 84 | 34 | 17 | 41 | 10 | 40 |
| Power | .40 | 1.47 | 84 | 33 | 18 | 46 | 10 | 40 |
| Political activities | .36 | 1.91 | 86 | 36 | 19 | 34 | 0 | 33 |
| Attitudes towards immigrants | .41 | 2.06 | 84 | 33 | 16 | 30 | 9 | 42 |
| Feeling depressed | .33 | 1.48 | 87 | 37 | 17 | 46 | 5 | 24 |
| Subjective happiness | .24 | .89 | 91 | 52 | 27 | 66 | 8 | 17 |
| Feeling optimistic | .26 | .83 | 90 | 49 | 24 | 68 | 4 | 13 |
| Pessimistic world view | .49 | 2.00 | 81 | 26 | 13 | 32 | 6 | 38 |
| Trust in other people | .52 | 1.81 | 80 | 25 | 11 | 37 | 10 | 33 |
| Trust in political institutions | .55 | 2.28 | 78 | 25 | 12 | 25 | 11 | 44 |
| Relationship to neighbors | .23 | 1.05 | 91 | 55 | 28 | 60 | 6 | 19 |
| Relationship to other people | .23 | .86 | 91 | 54 | 28 | 67 | 3 | 14 |
| Trust in democratic rights | .26 | 1.34 | 90 | 48 | 23 | 50 | 4 | 19 |
| Trust in democratic processes | .23 | .91 | 91 | 52 | 30 | 65 | 3 | 15 |
| Left-right scale | .16 | .95 | 94 | 68 | 39 | 64 | 0 | 10 |
| Religiosity (17) | .53 | 2.44 | 79 | 27 | 16 | 22 | 19 | 72 |
| **Average** | .33 | 1.40 | 87 | 43 | 22 | 50 | 8 | 29 |

*Note*. PCR: Percentage of common responses, AE: absolute effect in percent. Med: Median, ≥ 90/95: Percentage of pairs with a PCR ≥ 90/95 percent. Numbers in brackets indicate the number of countries where similarities are smaller than differences.

***Gender***. The amount of similarity was large, with an average PCR of 95 (*range* = 87 – 100) and an average AE of .02 (*range* = .00 to .12). The smallest percentage of common scores was found for religion (PCR = 87), with women scoring higher than men (*M*s = 4.80 vs 3.84, *medians* = 5.17 vs 3.92; cf. Table S8).

***Income***. The amount of similarity was again large, with an average PCR of 95 (*range* = 86 – 99) and an average AE of 3% (*range* = 0% – 10%). The smallest PCR was found for *having a pessimistic world-view* (PCR = 60), with people from the lowest two income deciles reporting being more pessimistic about their future compared to the people from the highest two deciles (*M*s < 2.61 vs > 3.11, *medians* = 2.50 vs 3.25, with lower values indicating a stronger pessimistic world-view; cf. Table S9).

***Education***. The amount of similarity was again large, with an average PCR of 94 (*range* = 86 – 99) and an average AE of 5% (*range* = 0% – 10%). The smallest PCR was between *attitudes towards immigrants* and *having a pessimistic world-view* (69 and 66 percent), with the lowest educated having a more pessimistic world-view than the second highest educated (lower tertiary educated; *M*s = 2.56 vs 3.16, *medians* = 2.50 vs 3.25), and the lowest educated having less favorable attitudes towards immigrants than the highest educated (*M*s = 4.23 vs 6.01, *medians* = 4.17 vs 6.17; cf. Table S10).

***Age***. The amount of similarity was again large, with an average PCR of 94 (*range* = 84 – 99) and an average AE of 5% (*range* = 0% – 10%). The smallest PCR was for the value type *stimulation* (PCR = 52), with the two groups of elderly participants (those born in 1947 or before) valuing stimulation less than the youngest participants (those born in 1991 or later; *M*s > 3.85 vs 2.59, *medians* = 4.0 vs 2.5, where lower numerical values indicate higher importance; cf. Table S11).

**Comparisons between all categories***.* To identify the categories for which the similarities are the largest, repeated-measurement ANOVAs were conducted for PCR and AE separately, with the five categories as the repeated-measures factor across all the 24 variables. Because of violations of the sphericity assumption, the Greenhouse-Geisser correction was applied. The ANOVAs revealed significant differences between categories in the PCRs (*F*[2.61, 60.12] = 27.02, *p* < .001, $\eta_{p}^{2}$ = .54), and the AEs (*F*[3.51, 80.69] = 12.52, *p* < .001, $\eta_{p}^{2}$ = .35). Pairwise comparisons revealed that country differed consistently from the four other categories (all *p*s ≤ .001), indicating that countries are less similar to each other than are the other categories. For PCR, countries were between 6.88 and 8.21 lower than for the other categories. For AE, countries were between 3% and 5% higher than the other categories. Differences between the other six categories were mostly non-significant.

## Tables of Detailed Results

The following tables provide detailed results for each category, variable, and statistic.

Table S8

*Comparisons of the 10 value types and 14 other variables between gender*

|  | Cohen’s *d* | | PCR | | | | AE | |
| --- | --- | --- | --- | --- | --- | --- | --- | --- |
|  | Med *d* | Max. *d* | Med | ≥ .90 | ≥ .95 | Min | Med AE | Max AE |
| Security | .17 |  | 93 |  |  |  | 0 |  |
| Tradition | .14 |  | 95 |  |  |  | 0 |  |
| Conformity | .02 |  | 99 |  |  |  | 0 |  |
| Benevolence | .15 |  | 94 |  |  |  | 0 |  |
| Universalism | .12 |  | 95 |  |  |  | 0 |  |
| Self-direction | .12 |  | 95 |  |  |  | 10 |  |
| Stimulation | .20 |  | 92 |  |  |  | 10 |  |
| Hedonism | .17 |  | 93 |  |  |  | 0 |  |
| Achievement | .16 |  | 94 |  |  |  | 0 |  |
| Power | .18 |  | 93 |  |  |  | 0 |  |
| Political activities | .11 |  | 95 |  |  |  | 0 |  |
| Attitudes towards immigrants | .03 |  | 99 |  |  |  | 1 |  |
| Feeling depressed | .27 |  | 89 |  |  |  | 5 |  |
| Subjective happiness | .17 |  | 93 |  |  |  | 8 |  |
| Feeling optimistic | .11 |  | 96 |  |  |  | 0 |  |
| Pessimistic world view | .13 |  | 95 |  |  |  | 6 |  |
| Trust in other people | .01 |  | 100 |  |  |  | 0 |  |
| Trust in political institutions | .04 |  | 99 |  |  |  | 0 |  |
| Relationship to neighbors | .03 |  | 99 |  |  |  | 0 |  |
| Relationship to other people | .10 |  | 96 |  |  |  | 2 |  |
| Trust in democratic rights | .05 |  | 98 |  |  |  | 1 |  |
| Trust in democratic processes | .07 |  | 97 |  |  |  | 2 |  |
| Left-right scale | .05 |  | 98 |  |  |  | 0 |  |
| Religiosity | .32 |  | 87 |  |  |  | 12 |  |
| **Average** | .12 |  | 95 |  |  |  | 2 |  |

*Note*. PCR: Percentage of common responses, AE: absolute effect in percent. Med: Median, ≥ 90/95: Percentage of pairs with a PCR ≥ 90/95 percent.

Table S9

*Comparisons of the 10 value types and 14 other variables between income groups*

|  | Cohen’s *d* | | PCR | | | | AE | |
| --- | --- | --- | --- | --- | --- | --- | --- | --- |
|  | Med *d* | Max. *d* | Med | ≥ .90 | ≥ .95 | Min | Med AE | Max AE |
| Security | .13 | .38 | 95 | 87 | 49 | 85 | 0 | 10 |
| Tradition | .19 | .50 | 93 | 64 | 33 | 80 | 10 | 20 |
| Conformity | .11 | .30 | 96 | 93 | 58 | 88 | 10 | 10 |
| Benevolence | .02 | .06 | 99 | 100 | 100 | 98 | 0 | 0 |
| Universalism | .02 | .07 | 99 | 100 | 100 | 97 | 0 | 0 |
| Self-direction | .12 | .35 | 95 | 93 | 51 | 86 | 0 | 10 |
| Stimulation | .12 | .36 | 95 | 93 | 51 | 86 | 0 | 10 |
| Hedonism | .13 | .38 | 95 | 80 | 49 | 85 | 0 | 10 |
| Achievement | .06 | .29 | 97 | 98 | 73 | 88 | 0 | 10 |
| Power | .07 | .25 | 97 | 100 | 78 | 90 | 0 | 0 |
| Political activities | .16 | .36 | 94 | 78 | 44 | 86 | 0 | 0 |
| Attitudes towards immigrants | .19 | .49 | 92 | 67 | 38 | 81 | 4 | 13 |
| Feeling depressed | .25 | .82 | 90 | 51 | 20 | 68 | 5 | 14 |
| Subjective happiness | .18 | .67 | 93 | 62 | 33 | 74 | 8 | 17 |
| Feeling optimistic | .18 | .58 | 93 | 62 | 33 | 77 | 0 | 4 |
| Pessimistic world view | .36 | 1.06 | 86 | 33 | 9 | 60 | 6 | 19 |
| Trust in other people | .20 | .57 | 92 | 64 | 33 | 78 | 3 | 13 |
| Trust in political institutions | .18 | .48 | 93 | 69 | 36 | 81 | 4 | 12 |
| Relationship to neighbors | .02 | .12 | 99 | 100 | 100 | 95 | 0 | 3 |
| Relationship to other people | .13 | .40 | 95 | 80 | 47 | 84 | 2 | 3 |
| Trust in democratic rights | .04 | .14 | 98 | 100 | 93 | 95 | 1 | 4 |
| Trust in democratic processes | .08 | .23 | 97 | 100 | 73 | 91 | 2 | 3 |
| Left-right scale | .08 | .27 | 97 | 98 | 71 | 89 | 0 | 0 |
| Religiosity | .19 | .47 | 92 | 60 | 33 | 82 | 8 | 20 |
| **Average** | .13 | .40 | 95 | 81 | 54 | 84 | 3 | 9 |

*Note*. PCR: Percentage of common responses, AE: absolute effect in percent. Med: Median, ≥ 90/95: Percentage of pairs with a PCR ≥ 90/95 percent.

Table 7

*Comparisons of the 10 value types and 14 other variables between education groups*

|  | Cohen’s *d* | | PCR | | | | AE | |
| --- | --- | --- | --- | --- | --- | --- | --- | --- |
|  | Med *d* | Max. *d* | Med | ≥ .90 | ≥ .95 | Min | Med AE | Max AE |
| Security | .12 | .26 | 95 | 95 | 52 | 89 | 0 | 0 |
| Tradition | .19 | .49 | 92 | 71 | 29 | 81 | 10 | 20 |
| Conformity | .10 | .24 | 96 | 100 | 71 | 90 | 10 | 10 |
| Benevolence | .04 | .19 | 98 | 100 | 76 | 93 | 0 | 0 |
| Universalism | .08 | .19 | 97 | 100 | 67 | 92 | 0 | 0 |
| Self-direction | .23 | .54 | 91 | 57 | 24 | 79 | 10 | 10 |
| Stimulation | .16 | .55 | 94 | 67 | 48 | 79 | 10 | 20 |
| Hedonism | .14 | .48 | 94 | 81 | 48 | 81 | 10 | 20 |
| Achievement | .19 | .42 | 93 | 76 | 29 | 83 | 10 | 20 |
| Power | .12 | .30 | 95 | 90 | 57 | 88 | 0 | 0 |
| Political activities | .28 | .61 | 89 | 48 | 14 | 76 | 0 | 0 |
| Attitudes towards immigrants | .35 | .80 | 86 | 33 | 29 | 69 | 9 | 20 |
| Feeling depressed | .18 | .59 | 93 | 52 | 29 | 77 | 5 | 10 |
| Subjective happiness | .12 | .45 | 95 | 76 | 52 | 82 | 8 | 17 |
| Feeling optimistic | .19 | .48 | 93 | 71 | 29 | 81 | 0 | 4 |
| Pessimistic world view | .34 | .87 | 87 | 38 | 19 | 66 | 6 | 19 |
| Trust in other people | .21 | .48 | 92 | 62 | 33 | 81 | 3 | 13 |
| Trust in political institutions | .15 | .43 | 94 | 71 | 38 | 83 | 4 | 11 |
| Relationship to neighbors | .07 | .19 | 97 | 100 | 76 | 92 | 3 | 3 |
| Relationship to other people | .13 | .32 | 95 | 90 | 52 | 87 | 1 | 3 |
| Trust in democratic rights | .03 | .17 | 99 | 100 | 71 | 93 | 1 | 3 |
| Trust in democratic processes | .18 | .55 | 93 | 62 | 19 | 78 | 3 | 10 |
| Left-right scale | .02 | .05 | 99 | 100 | 100 | 98 | 0 | 0 |
| Religiosity | .12 | .51 | 95 | 71 | 52 | 80 | 5 | 21 |
| **Average** | .16 | .42 | 94 | 75 | 46 | 83 | 5 | 10 |

*Note*. PCR: Percentage of common responses, AE: absolute effect in percent. Med: Median, ≥ 90/95: Percentage of pairs with a PCR ≥ 90/95 percent.

Table S11

*Comparisons of the 10 value types and 14 other variables between age groups*

|  | Cohen’s *d* | | PCR | | | | AE | |
| --- | --- | --- | --- | --- | --- | --- | --- | --- |
|  | Med *d* | Max. *d* | Med | ≥ .90 | ≥ .95 | Min | Med AE | Max AE |
| Security | .08 | .28 | 97 | 98 | 78 | 89 | 0 | 0 |
| Tradition | .23 | .65 | 91 | 56 | 22 | 74 | 10 | 20 |
| Conformity | .16 | .56 | 94 | 69 | 36 | 78 | 10 | 10 |
| Benevolence | .03 | .08 | 99 | 100 | 100 | 97 | 0 | 0 |
| Universalism | .06 | .26 | 98 | 98 | 80 | 90 | 0 | 7 |
| Self-direction | .15 | .47 | 94 | 78 | 44 | 81 | 10 | 10 |
| Stimulation | .41 | 1.29 | 84 | 31 | 9 | 52 | 10 | 30 |
| Hedonism | .34 | 1.07 | 86 | 38 | 13 | 59 | 10 | 30 |
| Achievement | .32 | .90 | 87 | 38 | 16 | 65 | 10 | 20 |
| Power | .22 | .62 | 91 | 58 | 24 | 76 | 10 | 20 |
| Political activities | .08 | .31 | 97 | 89 | 69 | 88 | 0 | 0 |
| Attitudes towards immigrants | .14 | .48 | 95 | 78 | 44 | 81 | 3 | 12 |
| Feeling depressed | .15 | .53 | 94 | 80 | 42 | 79 | 5 | 10 |
| Subjective happiness | .17 | .53 | 93 | 73 | 40 | 79 | 8 | 8 |
| Feeling optimistic | .10 | .38 | 96 | 89 | 60 | 85 | 0 | 4 |
| Pessimistic world view | .16 | .51 | 94 | 78 | 40 | 80 | 6 | 6 |
| Trust in other people | .03 | .12 | 99 | 100 | 100 | 95 | 0 | 3 |
| Trust in political institutions | .05 | .33 | 98 | 82 | 80 | 87 | 1 | 7 |
| Relationship to neighbors | .11 | .33 | 96 | 93 | 58 | 87 | 3 | 8 |
| Relationship to other people | .05 | .14 | 98 | 100 | 96 | 95 | 1 | 3 |
| Trust in democratic rights | .07 | .23 | 97 | 100 | 82 | 91 | 1 | 4 |
| Trust in democratic processes | .06 | .28 | 97 | 89 | 64 | 89 | 2 | 5 |
| Left-right scale | .05 | .14 | 98 | 100 | 96 | 94 | 0 | 0 |
| Religiosity | .19 | .61 | 93 | 64 | 38 | 76 | 8 | 24 |
| **Average** | .14 | .46 | 94 | 78 | 55 | 82 | 5 | 10 |

*Note*. PCR: Percentage of common responses, AE: absolute effect in percent. Med: Median, ≥ 90/95: Percentage of pairs with a PCR ≥ 90/95 percent.

# References

Barranti, M., Carlson, E. N., & Côté, S. (2017). How to test questions about similarity in personality and social psychology research Description and empirical demonstration of response surface analysis. *Social Psychological and Personality Science*, *8*(4), 465–475. https://doi.org/10.1177/1948550617698204

Bliese, P. D. (2000). Within-group agreement, non-independence, and reliability: Implications for data aggregation and Analysis. In K. J. Klein & S. W. Kozlowski (Eds.), *Multilevel Theory, Research, and Methods in Organizations* (pp. 349–381). San Francisco, CA: Jossey-Bass, Inc.

Cribbie, R. A., Gruman, J. A., & Arpin-Cribbie, C. A. (2004). Recommendations for applying tests of equivalence. *Journal of Clinical Psychology*, *60*(1), 1–10. https://doi.org/10.1002/jclp.10217

Davidov, E., & Meuleman, B. (2012). Explaining Attitudes Towards Immigration Policies in European Countries: The Role of Human Values. *Journal of Ethnic and Migration Studies*, *38*(5), 757–775. https://doi.org/10.1080/1369183X.2012.667985

Edwards, J. R. (2002). Alternatives to difference scores: Polynomial regression analysis and response surface methodology. In F. Drasgow & N. W. Schmitt (Eds.), *Advances in measurement and data analysis* (pp. 350–400). San Francisco, CA: Jossey-Bass.

Edwards, J. R., & Cable, D. M. (2009). The value of value congruence. *Journal of Applied Psychology*, *94*(3), 654–677. https://doi.org/10.1037/a0014891

Fischer, R., & Schwartz, S. (2011). Whence differences in value priorities? Individual, cultural, or artifactual sources. *Journal of Cross-Cultural Psychology*, *42*(7), 1127–1144. https://doi.org/10.1177/0022022110381429

Grönlund, K., & Setala, M. (2012). In honest officials we trust: Institutional confidence in Europe. *The American Review of Public Administration*, *42*(5), 523–542. https://doi.org/10.1177/0275074011412946

Henrich, J., McElreath, R., Barr, A., Ensminger, J., Barrett, C., Bolyanatz, A., … Ziker, J. (2006). Costly punishment across human societies. *Science*, *312*(5781), 1767–1770. https://doi.org/10.1126/science.1127333

Lakens, D. (2017). Equivalence tests: A practical primer for t tests, correlations, and meta-analyses. *Social Psychological and Personality Science*, 1948550617697177. https://doi.org/10.1177/1948550617697177

Lalonde, R. N., Cila, J., Lou, E., & Cribbie, R. A. (2015). Are we really that different from each other? The difficulties of focusing on similarities in cross-cultural research. *Peace and Conflict: Journal of Peace Psychology*, *21*(4), 525–534. https://doi.org/10.1037/pac0000134

Marozzi, M. (2014). Measuring trust in European public institutions. *Social Indicators Research*, 1–17. https://doi.org/10.1007/s11205-014-0765-9

McGraw, K. O., & Wong, S. P. (1992). A common language effect size statistic. *Psychological Bulletin*, *111*, 361–365. https://doi.org/10.1037/0033-2909.111.2.361

Morris, M. W. (2014). Values as the essence of culture foundation or fallacy? *Journal of Cross-Cultural Psychology*, *45*(1), 14–24. https://doi.org/10.1177/0022022113513400

Morris, M. W., Chiu, C., & Liu, Z. (2015). Polycultural psychology. *Annual Review of Psychology*, *66*(1), 631–659. https://doi.org/10.1146/annurev-psych-010814-015001

Ruscio, J. (2008). A probability-based measure of effect size: Robustness to base rates and other factors. *Psychological Methods*, *13*(1), 19–30. https://doi.org/10.1037/1082-989X.13.1.19

Ruscio, J., & Mullen, T. (2012). Confidence Intervals for the Probability of Superiority Effect Size Measure and the Area Under a Receiver Operating Characteristic Curve. *Multivariate Behavioral Research*, *47*(2), 201–223. https://doi.org/10.1080/00273171.2012.658329

Schmid, F., & Schmidt, A. (2006). Nonparametric estimation of the coefficient of overlapping—theory and empirical application. *Computational Statistics & Data Analysis*, *50*(6), 1583–1596. https://doi.org/10.1016/j.csda.2005.01.014

Schwartz, S. H. (1992). Universals in the content and structure of values: Theoretical advances and empirical tests in 20 countries. *Advances in Experimental Social Psychology*, *25*, 1–65.

Schwartz, S. H. (2003). Chapter 7: A Proposal for measuring value orientations across nations. Retrieved from http://www.europeansocialsurvey.org/docs/methodology/core_ess_questionnaire/ESS_core_questionnaire_human_values.pdf

Schwartz, S. H., Melech, G., Lehmann, A., Burgess, S., Harris, M., & Owens, V. (2001). Extending the cross-cultural validity of the theory of basic human values with a different method of measurement. *Journal of Cross-Cultural Psychology*, *32*(5), 519–542. https://doi.org/10.1177/0022022101032005001

Visser, M., Scholte, M., & Scheepers, P. (2013). Fear of crime and feelings of unsafety in European countries: Macro and micro explanations in cross-national perspective. *The Sociological Quarterly*, *54*(2), 278–301. https://doi.org/10.1111/tsq.12020
